# Supplementary material for: The K219T-Lamin mutation induces conduction defects through epigenetic inhibition of SCN5A in human cardiac laminopathy
Source: Nat Commun. 2019 May 22;10:2267. doi: 10.1038/s41467-019-09929-w (PMC6531493; doi:10.1038/s41467-019-09929-w)
Supplement: Supplementary file 1 — Supplementary Information [file 41467_2019_9929_MOESM1_ESM.pdf]

The K219T-Lamin mutation induces conduction defects through epigenetic inhibition of *SCN5A* in human cardiac laminopathy

Salvarani*, Crasto* et al

SUPPLEMENTARY INFORMATION

Supplementary Figures and Legends

Figure S1

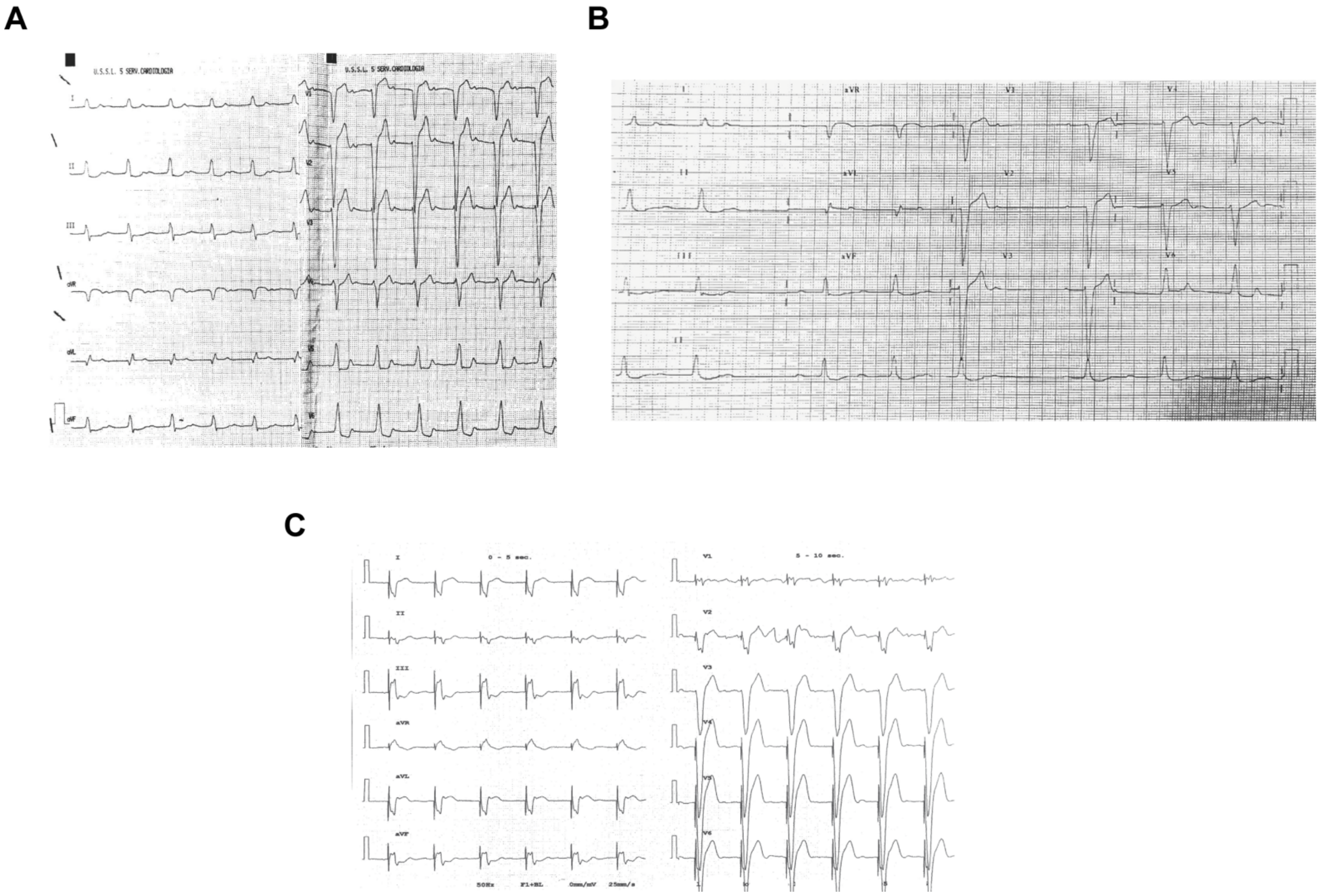

Supplementary Figure 1 – Electrocardiogram (ECG) traces. (A, B, C) ECG traces relative to LMNA #2 patient showing the progressive conduction block defects. In A, ECG traces show sinus rhythm, first degree atrio-ventricular block (P-R interval 440 ms) and left bundle branch block (LBBB). Traces in B evidence sinus rhythm and second degree atrio-ventricular block. In C, atrial fibrillation is appreciable with ventricular induced induced pacing rhythm.

Figure S2

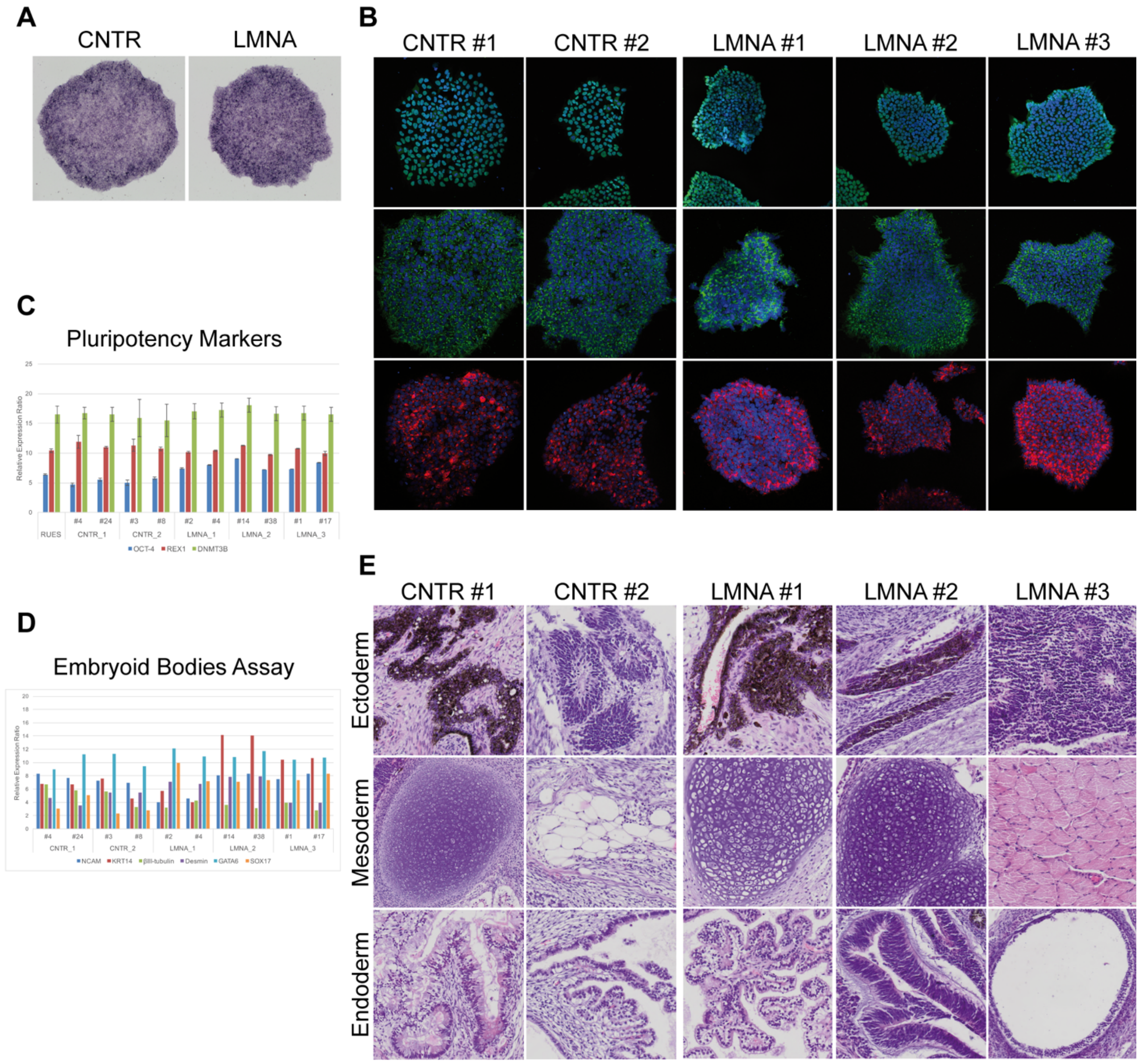

Supplementary Figure 2 - Characterization of iPSC lines. (A) Representative images of alkaline phosphatase activity in CNTR- and K219T-iPSC lines. **(B)** Immunofluorescence staining showing the expression of Oct4 (green) and the surface pluripotency markers SSEA4 (green) and TRA1-60 (red) in the generated lines. One representative image per subject is shown. Nuclei were stained with DAPI. **(C)** Realtime PCR for *Oct4*, *Rex1* and *Dnmt3B*, showing induction of expression of the three markers of pluripotency in two lines per subject from whom lines were generated. Data are relative to parental fibroblasts and were normalized using HPRT as a housekeeping gene. cDNA from the RUES2 embryonic stem cell line was used as a positive control. The graph is relative to one

representative experiment (out of three). **(D)** In vitro differentiation through the formation of embryoid bodies (EBs) indicated that the generated lines were able to differentiate into cells expressing markers of the three germ layers. The graph shows the results of realtime PCR for markers of ectoderm (NCAM, KRT14, and III-tubulin), mesoderm (desmin) and endoderm (GATA6 and SOX17) in EBs at 30 days of differentiation obtained from all the lines used for the experiments. Data are relative to undifferentiated iPSCs and were normalized to the housekeeping gene *HPRT* and to 18S. **(E)** Teratoma formation assay demonstrating the ability of the generated lines to differentiate in vivo into tissues originating from ectoderm (neural rosettes, retinal epithelium), mesoderm (cartilage, adipose tissue and muscle) and endoderm (intestinal or respiratory epithelium). One representative image per subject is shown.

Figure S3

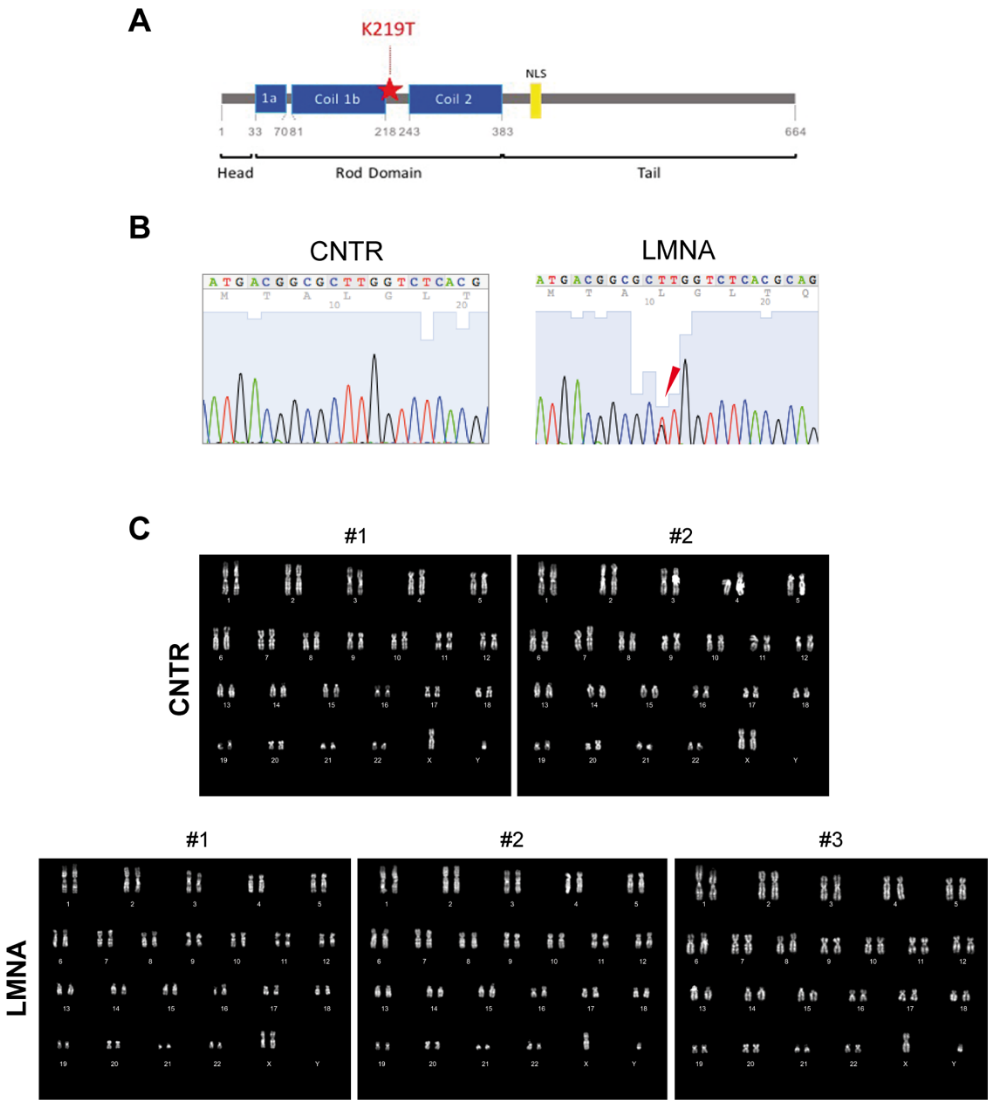

Supplementary Figure 3 - Genetic analyses. (A) Schematic representation of the Lamin A protein structure, showing the localization of the *p.*K219T mutation mainly investigated in this study. **(B)** Representative electropherograms from DNA sequencing of exon 4 of *LMNA* showing that the generated LMNA-iPSC lines carried the alanine-to-cytosine transversion at position 656 (c.656A<C) in heterozygosity as in the patients (the reverse sequence is shown in the figure; the T<C mutation is indicated by the arrow), whereas CNTR iPSCs were wild-type. The nucleotide mutation corresponds to the amino acid change *p.*K219T. RefSeq LMNA: NM_170707.3. **(C)** Karyotype analyses confirming that the employed iPSC lines did not accumulate any major genetic abnormality during reprogramming. One representative image per subject is shown.

Figure S4

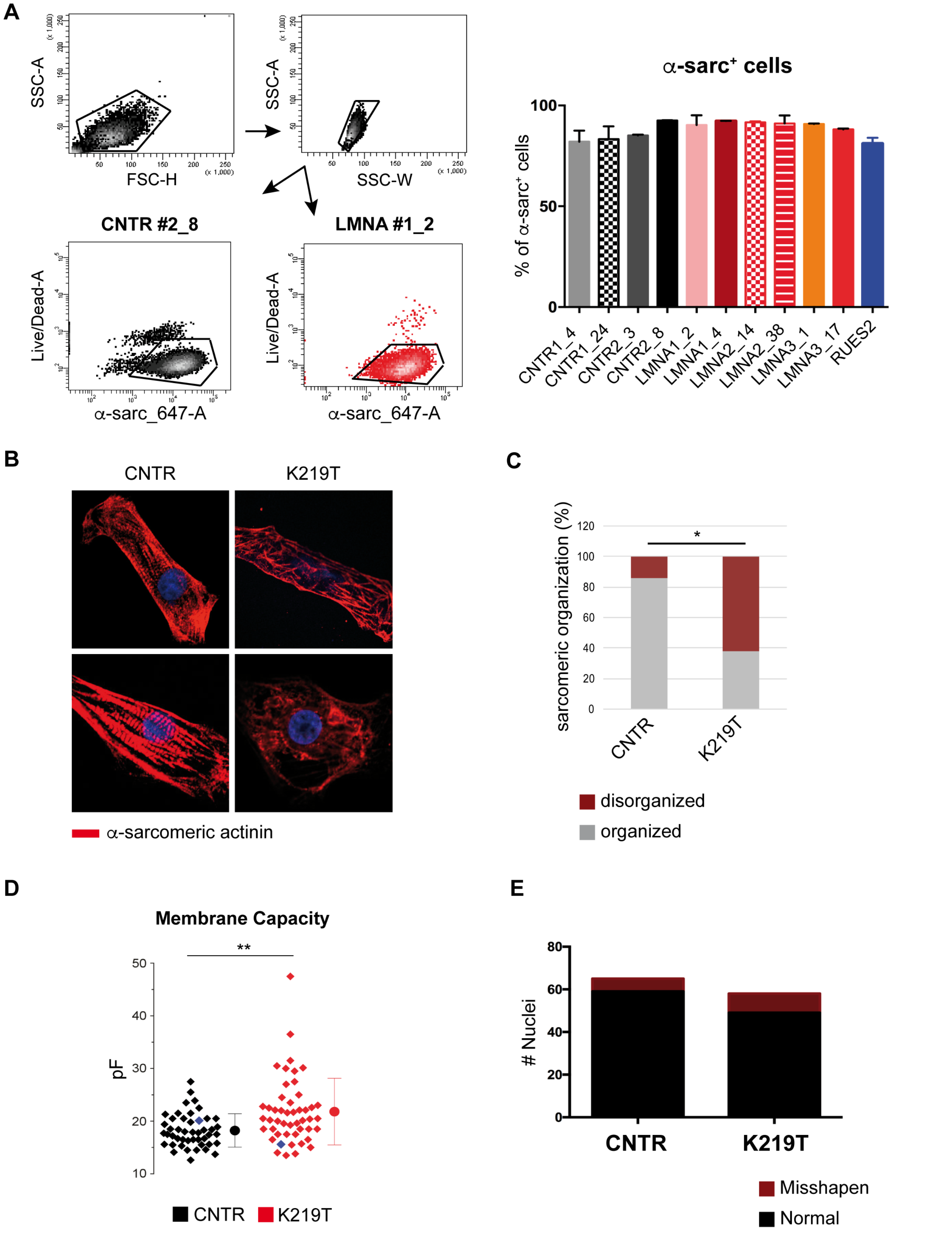

Supplementary Figure 4 – K219T-CMs recapitulate phenotypic traits of dilated cardiomyopathy. (A) Flow cytometry analysis of iPSC-CMs. Left: representative flow cytometry

plots of human iPSC-CMs. Debris were excluded and cells were selected based on size (upper left panel). After exclusion of doublets (upper right panel) and dead cells, single live cells were analysed using a cardiac-specific antibody against sarcomeric α-actinin. Scatter plots and gates of a representative experiments in CNTR- and K219T-LMNA lines are shown in the bottom panels. Right: percentage of sarcomeric α-actinin$^+$ cells. Histograms depict all clones analysed in duplicate. **(B)** α-Sarcomeric actinin staining of CMs differentiated from CNTR- and K219T-LMNA-iPSCs revealed sarcomeric abnormalities (fragmented and punctuated staining) in the latter. Nuclei were stained with DAPI. **(C)** Bar graph of the percentages of cells displaying sarcomeric disorganization in CNTR- and K219T-CMs. CNTR-CMs, n=83; K219T-CMs, n=126. **(D)** Patch-clamp recordings of membrane capacity in CNTR-CMs and K219T-CMs showing a higher mean value in the latter. CNTR-CMs, n=46; K219T-CMs, n=48. **(E)** Analysis of nuclear abnormalities in CNTR- and K219T-CMs, showing no significant differences in the number of misshapen nuclei between the two. All values are reported as means ± SD. *$p < 0.05$; **$p < 0.005$ (unpaired t-test).

Figure S5

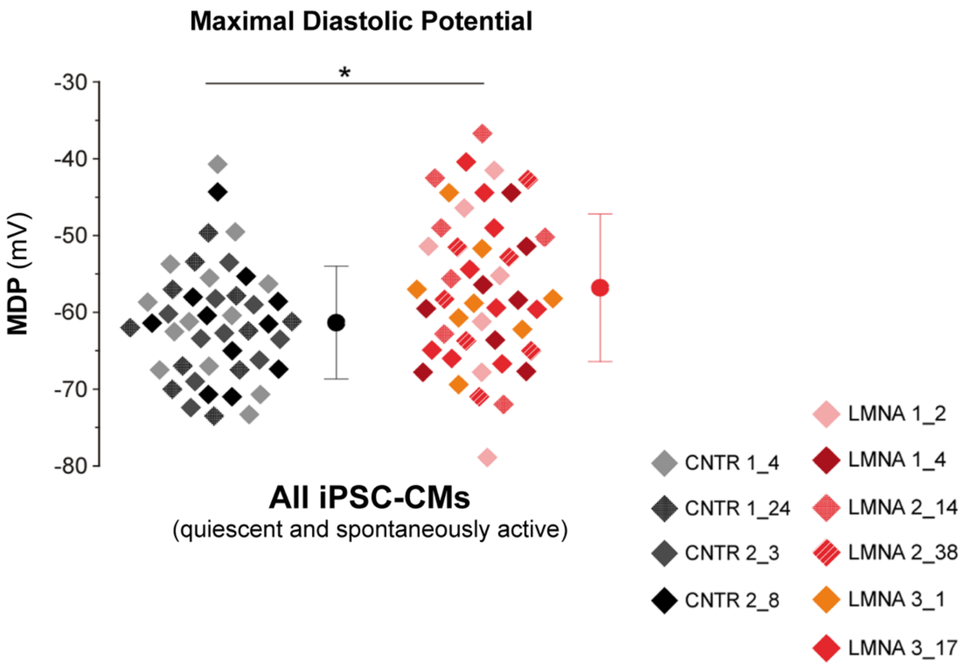

Supplementary Figure 5 – Patch-clamp recordings of resting membrane potential in individual cell lines. Dot plot of maximal diastolic potential (MDP) data measured from all iPSC-CMs, showing the independent distribution of four CNTR- and six K219T-LMNA clones. Of note, we show MDP data for CMs from the individual cell lines because MDP represents the electrophysiological recording from which all the other AP parameters are measured. Cumulated values are reported as means ± SD. *$p < 0.05$ (unpaired t-test).

Figure S6

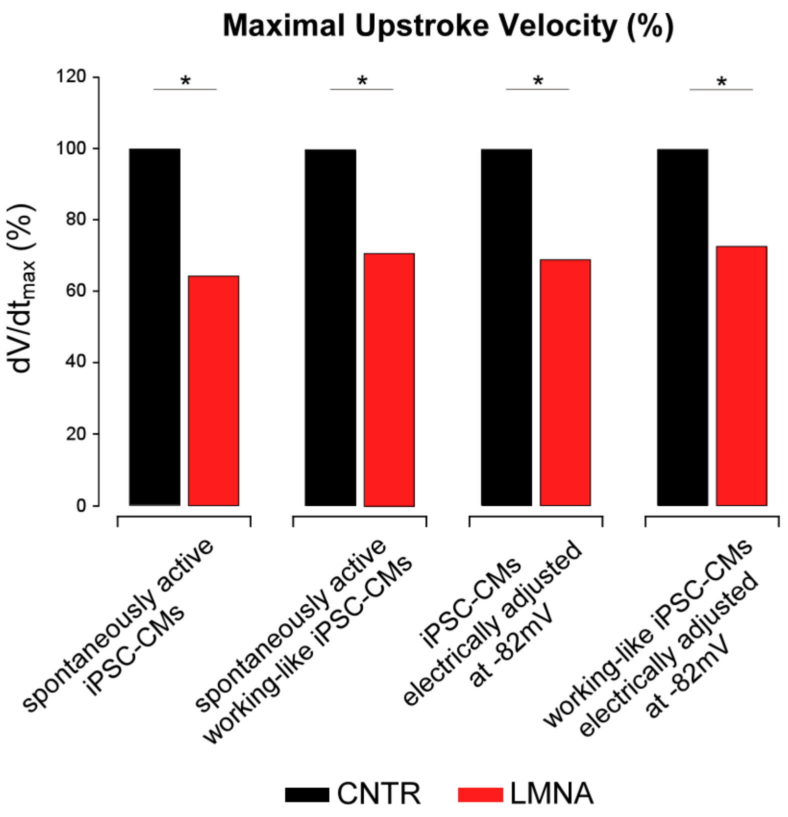

Supplementary Figure 6 - Assessment of *dV/dt_{max}* changes in different populations of iPSC-derived CMs. Maximal upstroke velocity (values from spontaneously active iPSC-CMs, working-like iPSC-CMs, and iPSC-CMs electrically adjusted to ~ -82mV) in K219T-LMNA-CMs relative to CNTR-CMs, expressed as a percentage. Values are reported as means ± SD. *$p < 0.05$ (unpaired t-test).

Figure S7

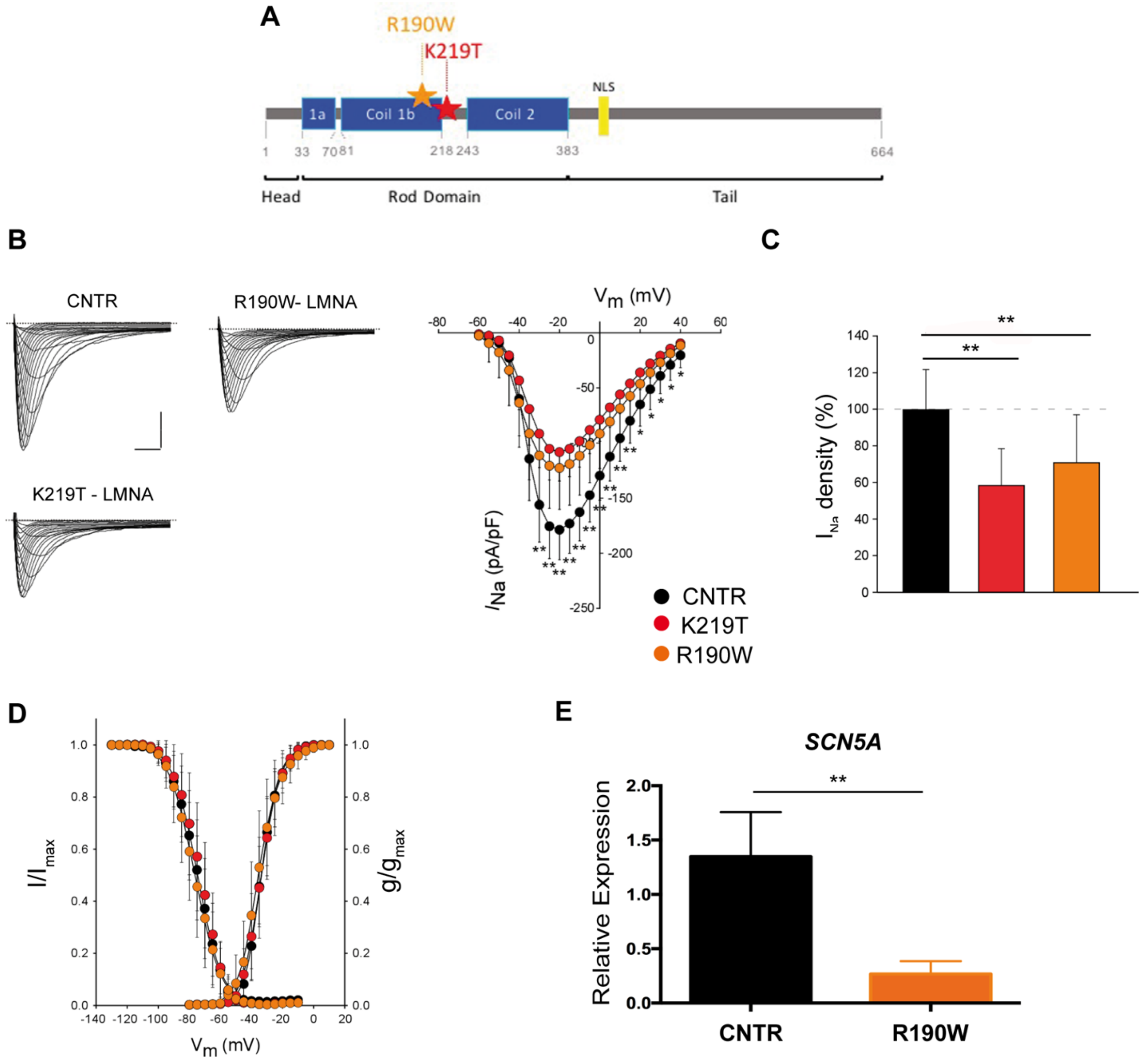

Supplementary Figure 7 - The R190W Lamin A/C mutation is also associated with reduced peak sodium currents and diminished *SCN5A* gene expression. (A) Lamin A protein structure showing localization of the R190W and K219T mutations in the Rod domain of the protein. **(B)** Left column: examples of Na^+ current (I_{Na}) traces recorded in CNTR- (top), R190W-LMNA (top) and K219T-LMNA (bottom) CMs (scale bar, 2ms; 50pA/pF). Right panel: *I-V* curves constructed from average peak sodium current density as a function of voltage command measured in the three conditions described above, showing significant sodium current reductions in CMs carrying the R190W or K219T mutation (CNTR: n=23; R190W: n=18; K219T: n= 22). **(C)** I_{Na} density, measured at -30 mV, in R190W-CMs (110.93 ± 40.61 pA/pF) relative to K219T-CMs (91.58 ± 30.69 pA/pF)

and CNTR-CMs (156.12 ± 33.73 pA/pF), expressed as a percentage. Values for K219T- (red bar) and CNTR- (black bar) CMs are reported from Fig. 2B as references for evaluating the relative effect of the R190W mutation. **(D)** Voltage dependences: steady state activation (CNTR: n=23; R190W: n=18; K219T: n= 22)/inactivation (CNTR: n=18; R190W: n=14; K219T: n= 17) curves. **(E)** Realtime PCR showing *SCN5A* expression is reduced in CMs generated from iPSCs carrying the R190W mutation. Data are represented relative to CNTR-CMs and normalized to expression of the housekeeping genes *18S* and *HGPRT*. All values are reported as means ± SD. *$p < 0.05$; **$p < 0.005$ (unpaired t-test).

Figure S8

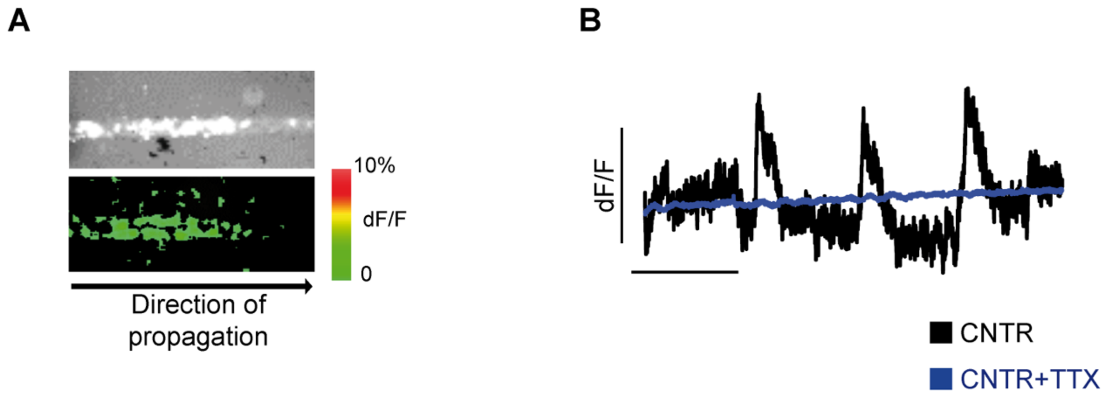

Supplementary Figure 8 – Pharmacological blockage of $Na_v1.5$ with tetrodotoxin (TTX) abolishes generation and propagation of optical action potential in iPSC-CMs. (A) Representative images of a monolayer of iPSC-CMs in geometrically defined strands (80-µm wide, 1 cm long). Top: phase contrast image of a portion of the strands observed in the field of view; Bottom: same strand during impulse propagation from left to right, following electrical stimulation. **(B)** Stimulated optical action potentials in monolayers of CNTR (black) and CNTR in the presence of 100 µmol/L TTX in the perfused solution (red). n=17 for CNTR and n=30 for CNTR+TTX.

Figure S9

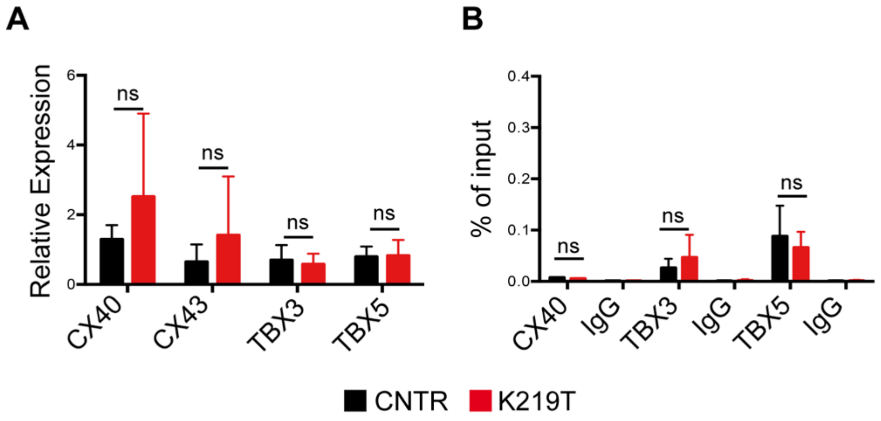

Supplementary Figure 9 - Epigenetic modulation of genes involved in cardiac conduction. (A) Realtime PCR for genes encoding proteins with key roles in conduction, namely Connexin-40 (*CX40*), Connexin-43 (*CX43*), T-box transcription factor-3 (*TBX3*) and T-box transcription factor-5 (*TBX5)*, revealed no significant differences in expression between CNTR-CMs and K219T-CMs. **(B)** ChIP for Lamin A/C followed by realtime PCR of the immunoprecipitated DNA, assessing the binding of Lamin A/C at the promoter region of the genes analysed in A. No significant enrichment was detectable at any of the analysed genes. *CX43* has not been included in the graph since values from realtime PCR were undetectable in both conditions. All values are reported as means ± SD. ns= not significant.

Figure S10

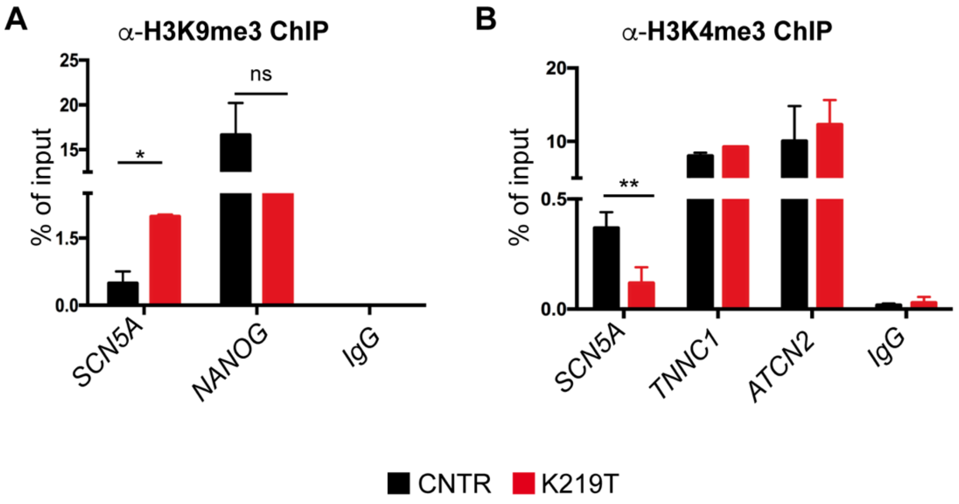

Supplementary Figure 10 – Additional analysis on epigenetic marks regulating *SCN5A* gene expression. (A) ChIP for H3K9me3, showing enrichment of this repressive mark at the promoter region of *SCN5A* in K219T-CMs. The mark was equally enriched on the *NANOG* promoter region of CNTR- and K219T-CMs, used as control. Data are presented as percentage of input chromatin precipitated. **(B)** ChIP for H3K4me3, showing enrichment of this active mark at the promoter region of *SCN5A* in CNTR-CMs, and thus supporting active transcription of this gene in control cells. Analysis of deposition of the H3K4me3 mark at *TNNC1* and *ACTN2* served as a control for the antibody. Data are presented as a percentage of input chromatin precipitated. All values are reported as means ± SD. *p<0.05; **p<0.005; ns=not significant (unpaired t-test).

Figure S11

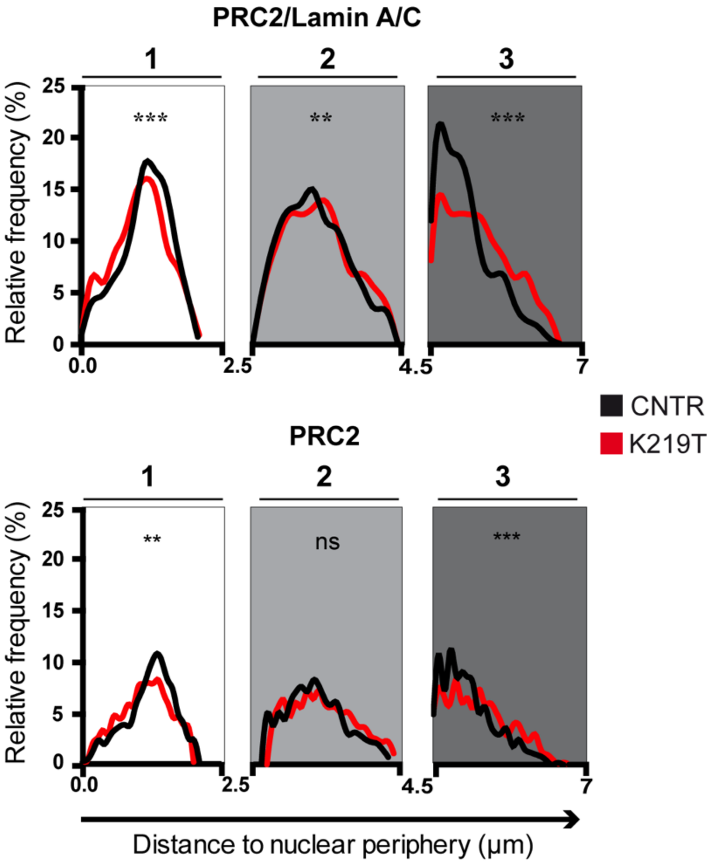

Supplementary Figure 11 – Distance distribution profiles of Lamin A/C and PRC2 in CNTR- and K219T-CM nuclei. Distribution profiles of PRC2-Lamin A/C aggregates (top) and PRC2 (bottom). Data are referred to 3D-STED microscopy images from CNTR- (black line) and K219T- (red line) CMs and show a significantly different dynamic of the two proteins in CNTR- and K219T-CMs, with a subpopulation of the PRC2/Lamin A/C aggregates in K219T-CMs (top) localized closer to the nuclear periphery (zone 1) than in CNTR-CMs, and another subpopulation relocated towards the nuclear interior (zone 3). Distribution relative to PRC2 itself shows profiles similar to those obtained from the colocalization signals above. The distances to the nuclear periphery were calculated relative to the "nuclear edge", identified as a ring of spots of Lamin A/C, and represented as frequency distribution profiles using cubic spline of histogram with 0.2 μm Bin. Relative distributions were evaluated in three selected zones: 1, periphery (white); 2, middle (light grey); and 3, central (dark grey). Values are median ± SD of 5 cells per condition (***$p<0.0001$; **$p<0.01$, two-way ANOVA).

Figure S12

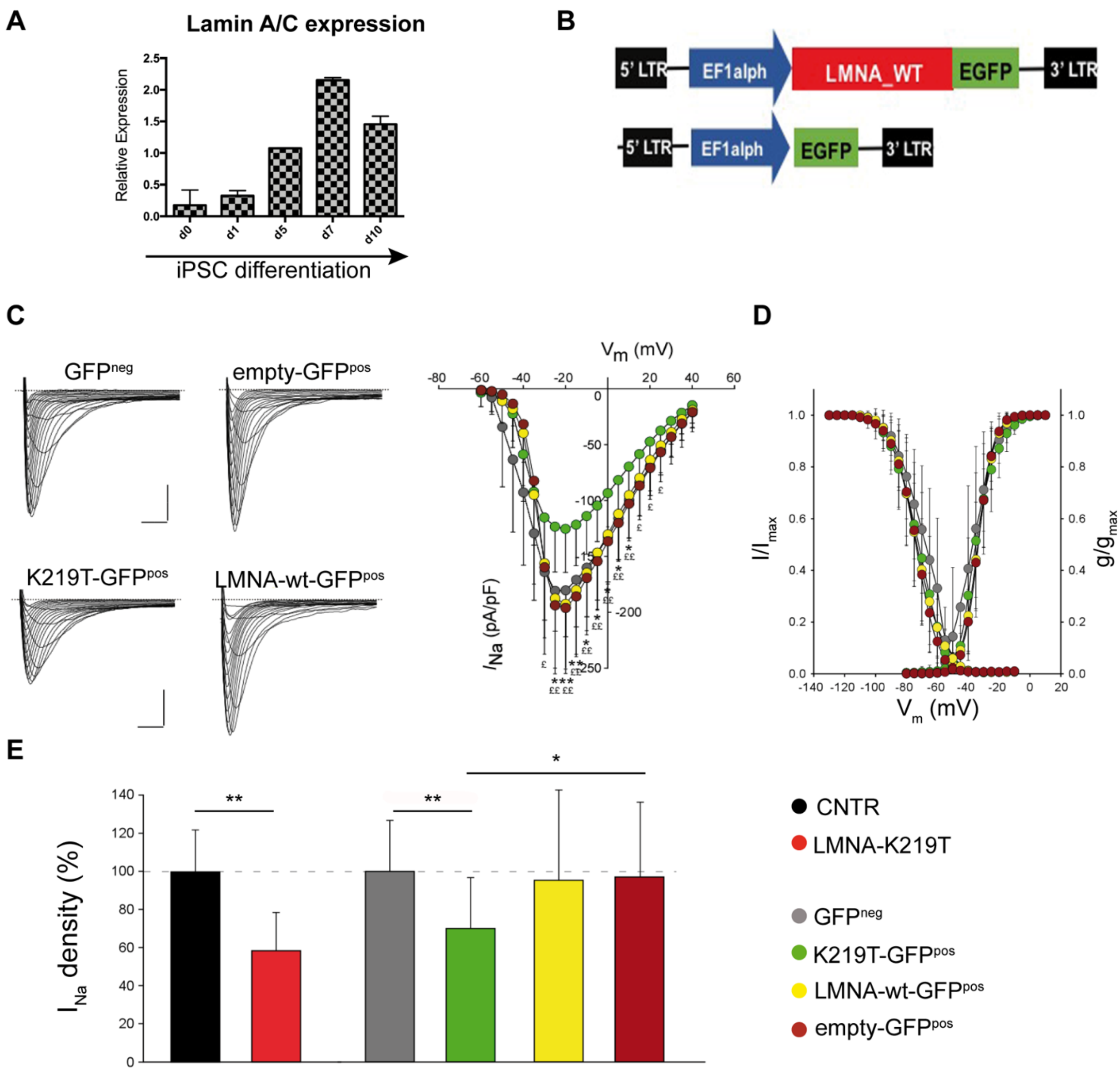

Supplementary Figure 12 – Additional control for LMNA overexpression experiments. (A) RT-PCR for Lamin A/C expression during differentiation of wild-type iPSC toward cardiomyocytes. **(B)** Additional lentiviral vectors used for the experiments encoding the C-terminal GFP-tagged wild- type *LMNA* gene (top) or GFP alone (pLEnti-GFP backbone). **(C)** Left panel: examples of Na^+ current (I_{Na}) traces recorded in GFP^{neg} and empty-GFP CMs (top); K219T-GFP^{pos} and LMNA-WT-GFP CMs (bottom), (scale bar, 2ms, 50pA/pF). Right panel: *I-V* curves constructed from average peak sodium current density as a function of voltage command measured in the four conditions described above (GFP^{neg}: n=14; K219T-GFP^{pos}: n=21; empty -GFP: n=15; LMNA-WT-GFP: n=14) showing

significant sodium current reductions in K219T-GFP^{pos} cells, while there were no changes compared to control cells (GFP^{neg}) induced by overexpression of either the empty-GFP vector or the one expressing wild-type *LMNA*. All values are reported as means ± SD. *p <0.05; **p <0.005 and $^{£}$p <0.05; $^{££}$p <0.005 vs K219T-GFP^{pos}. **(D)** Voltage dependences: steady state activation (GFP^{neg}: n=14; K219T-GFP^{pos}: n=21; empty-GFP: n=15; LMNA-WT-GFP: n=14)/inactivation (GFP^{neg}: n=11; K219T-GFP^{pos}: n=17; empty-GFP: n=12; LMNA-WT-GFP: n=12) curves. **(E)** I_{Na} density, measured at -30 mV, in K219T-CMs (91.58 ± 30.69 pA/pF) relative to CNTR-CMs (156.12 ± 33.73 pA/pF) and K219T-GFP^{pos} (115.20 ± 43.35 pA/pF), LMNA-WT-GFP CMs (156.22 ± 81.41 pA/pF) and empty-GFP CMs (159.76 ± 63.31 pA/pF) relative to GFP^{neg} CMs (164.02 ± 43.18 pA/pF) all expressed as a percentage. Values for K219T- (red bar) and CNTR- (black bar) CMs are reported from Fig. 2B as references for evaluating the relative effect in the analysed overexpression conditions. *p <0.05; **p <0.005 (unpaired t-test).

Figure S13

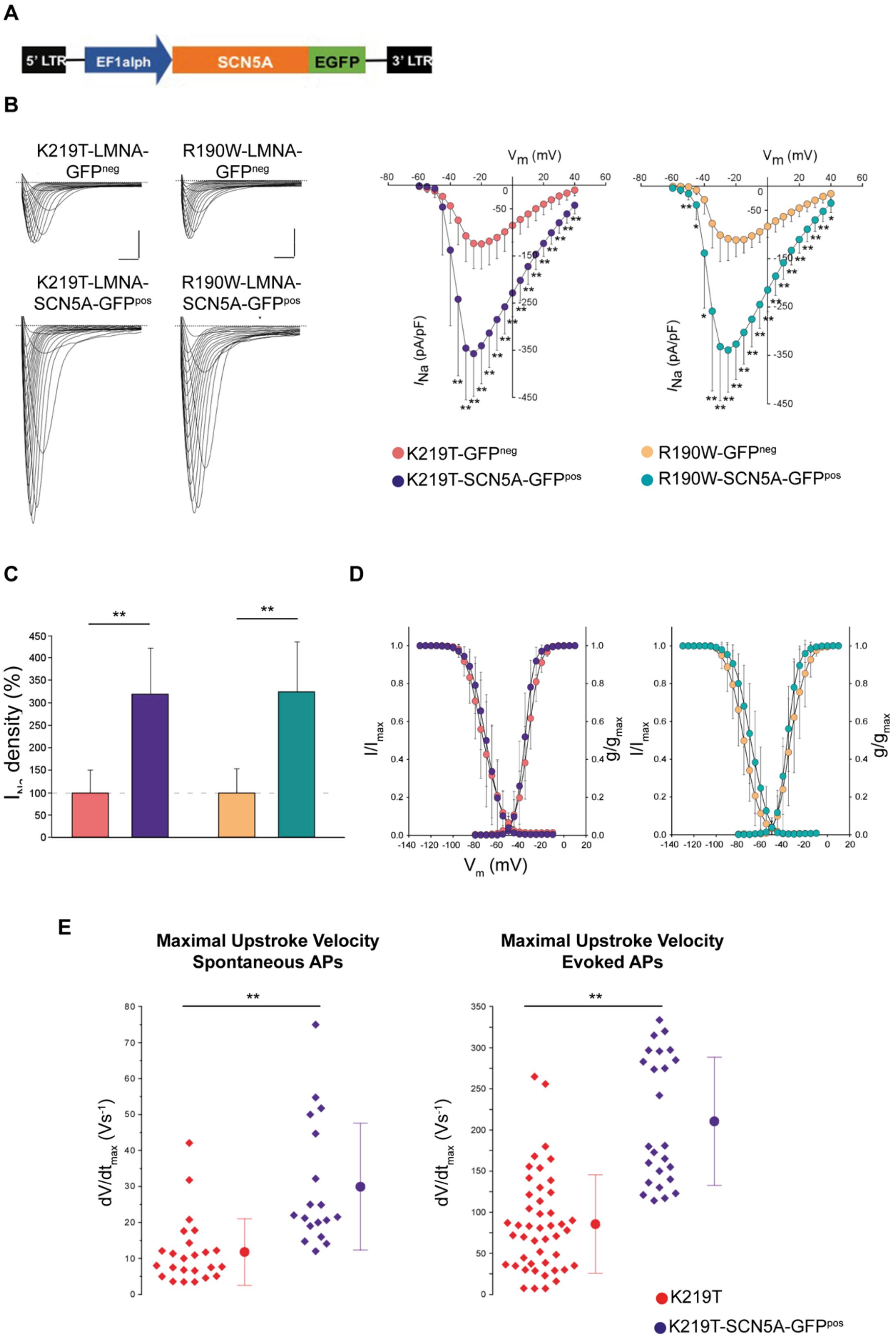

Supplementary Figure 13 – Overexpression of *SCN5A* rescues the functional phenotype in K219T-CMs. (A) Lentiviral vector expressing the C-terminal GFP-tagged *SCN5A* used to produce

the lentiviral particles for overexpression. **(B)** Left panel: examples of Na^+ current (I_{Na}) traces recorded in K219T-GFP^{neg} and R190W-GFP^{neg} CMs (top); K219T-SCN5A-GFP^{pos} and R190W-SCN5A-GFP^{pos} CMs (bottom), (scale bar, 2ms, 50pA/pF). Right panel: *I-V* curves constructed from average peak sodium current density as a function of voltage command measured in K219T-GFP^{neg} (n=6), K219T-SCN5A-GFP^{pos} (n=14), R190W-GFP^{neg} (n=6), and R190W-SCN5A-GFP^{pos} (n=13) CMs, showing significant sodium current increases in K219T-SCN5A-GFP^{pos} and R190W-SCN5A-GFP^{pos} cells compared to their respective GFP^{neg} counterpart. **p<0.005 vs K219T-GFP^{neg} and R190W-GFP^{neg} respectively. **(C)** I_{Na} density, measured at -30 mV, in K219T-SCN5A-GFP^{pos} CMs (345.42 ± 109.79 pA/pF) relative to K219T-GFP^{neg} CMs (107.52 ± 54.71 pA/pF) and R190W-SCN5A-GFP^{pos} CMs (331.86 ± 111.61 pA/pF) relative to R190W-GFP^{neg} CMs (101.38 ± 54.15 pA/pF) expressed as a percentage. **(D)** Voltage dependences: steady state activation (K219T-GFP^{neg}: n=6; K219T-SCN5A-GFP^{pos}: n=14; R190-GFP^{neg}: n=6; R190W-SCN5A-GFP^{pos}: n=13)/inactivation (K219T-GFP^{neg}: n=5; K219T-SCN5A-GFP^{pos}: n=12; R190W-GFP^{neg}: n=5; R190W-SCN5A-GFP^{pos}: n=11) curves. **(E)** Dot plot for the maximal upstroke velocity (dV/dt_{max}) measured from spontaneous (left panel) and evoked (right panel) action potentials (APs) in K219T-CMs, before and after administration of *SCN5A*, showing the increase of the maximal upstroke velocity in CMs overexpressing the gene (Spontaneous APs - K219T-CMs: n=24; K219T-SCN5A-GFP^{pos}: n=18. Evoked APs - K219T-CMs: n=46; K219T-SCN5A-GFP^{pos}: n=25). All values are reported as means ± SD. *p <0.05; **p <0.005 (unpaired t-test).

Figure S14

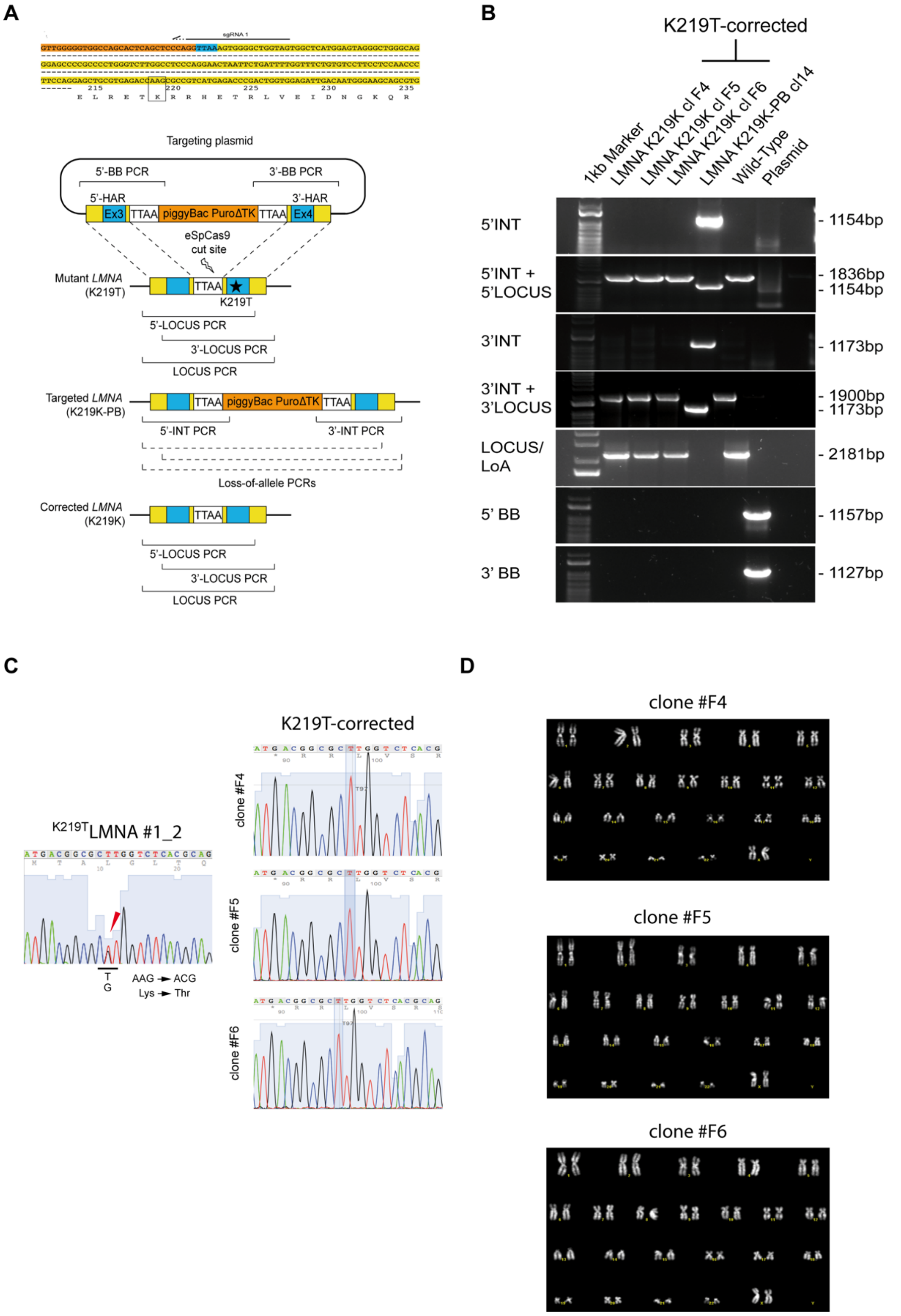

Supplementary Figure 14 - Generation and characterization of isogenic K219T-corrected iPSC lines. (A) Upper panel: representation of the endogenous "TTAA" site in the third intron of *LMNA* and located 135 bp upstream to the AC mutation in exon 4 leading to the K219T change, and of the

single guide RNA (sgRNa) sequence. The bottom panel gives a schematic view of the gene correction strategy, including the position of the PCRs used for the genotyping. **(B)** Genotyping PCRs for the three lines generated from the K219T LMNA #1_2 iPSC line. DNA from a control line and the targeting plasmid have been included as controls. **(C)** Electropherograms of the three isogenic K219T-corrected iPSC lines generated in this study, showing the specific correction of the mutation. Sequence of the parental K219T-LMNA iPSC line (LMNA #1_2) is shown on the left as a reference, with the heterozygous nucleotide change indicated by the arrow. **(D)** Karyotype analyses of the three isogenic K219T-corrected clones, confirming that no major genetic abnormalities were induced by the gene editing.

Figure S15

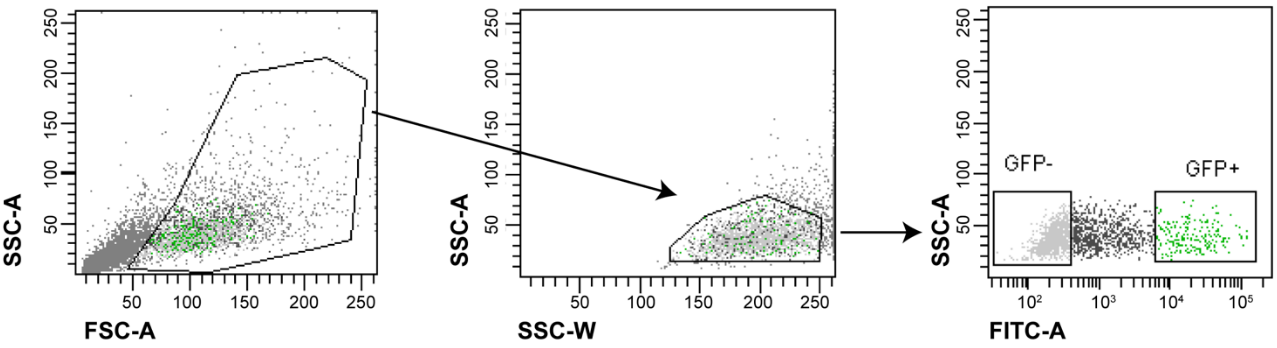

Supplementary Figure 15 – Sorting gating strategy. Sorting strategy and cell population selection relative to FACS sorting experiments represented in Figure 8E

Supplementary Tables and Videos

Patient #	LMNA #1	LMNA #2	LMNA #3	CNTR #1	CNTR #1
**Phenotype**	DCM	DCM	DCM	healthy	healthy
**Gender**	F	M	M	M	F
**Age** (years)	15	54	51	29	32
**Age at diagnosis** (years)	n.a.	42	39	n/a	n/a
***p*.K219T LMNA**	Y	Y	Y	N	N
***p*.L4855F TTN variant**	N	Y	Y	N	N
**Heart Transplant**	N	Y (2005)	Y (2006)	N	N
**Conduction abnormalities**	none	1st degree AV block , LBBB (05-2000), 2nd degree AV block (06-2000)	Atrial Flutter, 3rd degree AV block, left bundle branch (01-2000)	none	none
**History of spontaneous arrhythmias**	N	Y (Paroxismal AF)	Y (AF, atrial flutter, Sustained VT, VF)	N	N
**Abnormal IVS motion** (echo)	N	Y	Y	N	N
**PM** (year of implantation)	N	Y (PM 2000 ICD BIV 2003)	Y (PM 2000 ICD BIV 2005)	N	N
**EPS**	not performed	non inducible ventricular arrhythmias	non inducible ventricular arrhythmias	not performed	not performed

Supplementary Table 1 – Clinical information on patients and control family members

DCM, dilated cardiomyopathy; Y, yes; N, no; F, female; M, male; n.a., not available; n/a, not applicable; AF, atrial fibrillation; VT, ventricular tachycardia; VF, ventricular flutter; ECG, electrocardiogram; AV, atrio-ventricular; LBBB, left bundle branch block; IVS, interventricular septum; PM, pacemaker; ICD, implantable cardioverter defibrillator; BIV, biventricular; EPS, electrophysiological study (on explanted heart).

	dV/dt_{max} ($V\ s^{-1}$)	Overshoot (mV)	APA (mV)	MDP (mV)	RMP (mV)	APD_{90} (msec)	C_m (pF)
**Spontaneous APs**							
**CNTR**	18.4 ± 9.7* (n=33)	22.6 ±11.2** (n=33)	86.4 ± 15.2* (n=33)	-63.9 ± 5.6 (n=33)		316 ± 159 (n=33)	18.1 ± 3.0* (n=33)
**LMNA-K219T**	11.8 ± 9.2 (n=24)	12.3 ± 11.6 (n=24)	74.2 ± 16.1 (n=24)	-61.9 ± 7.0 (n=24)		285 ± 122 (n=24)	21.6 ± 7.7 (n=24)
**Evoked APs**							
**CNTR**	124.7 ± 71.6* (n=45)	24.4 ± 8.6** (n=45)	106.8 ± 9.2** (n=45)	-61.3 ± 7.4* (n=45)	-82.4 ± 2.4 (n=45)	222 ± 125* (n=45)	18.2 ± 3.2** (n=45)
**LMNA-K219T**	85.6 ± 60.1 (n=46)	14.9 ± 11.1 (n=46)	97.3 ± 11.5 (n=46)	-56.8 ± 9.6 (n=46)	-82.4 ± 2.4 (n=46)	165 ± 85 (n=46)	22.0 ± 6.3 (n=46)

Supplementary Table 2 - Characterization of spontaneous and evoked action potentials in iPSC-derived CMs. dV/dt_{max}, maximal upstroke velocity; Overshoot, peak voltage; APA, action potential amplitude; MDP, maximal diastolic potential; RMP, resting membrane potential induced by hyperpolarizing current input; APD_{90}, action potential duration measured at 90% repolarization; C_m, membrane capacity. * $P<0.05$, ** $P<0.005$ vs LMNA-K219T (Student's t-test for unpaired samples).

	dV/dt_{max} (V s^{-1})	Overshoot (mV)	APA (mV)	MDP (mV)	RMP (mV)	APD_{90} (msec)	C_m (pF)
**Spontaneous APs**							
**CNTR** *Working-like*	20.6 ± 8.8* (n=28)	26.1 ±7.5** (n=28)	90.6 ± 11.5* (n=28)	-64.5 ± 5.3 (n=28)		343 ± 158 (n=28)	17.7 ± 2.5* (n=28)
**LMNA-K219T** *Working-like*	14.5 ± 9.7 (n=17)	17.1 ± 10.2 (n=17)	81.3 ± 13.4 (n=17)	-64.3 ± 6.6 (n=17)		324 ± 123 (n=17)	20.4 ± 4.5 (n=17)
**CNTR** *Pacemaker-like*	4.0 ± 0.4 (n=3)	1.4 ±9.7 (n=3)	57.3 ± 12.1 (n=3)	-55.8 ± 2.4 (n=3)		186 ± 74.3 (n=3)	20.7 ± 6.7 (n=3)
**LMNA-K219T** *Pacemaker-like*	4.1 ± 0.7 (n=5)	0.3 ± 3.5 (n=5)	55.2 ± 13.4 (n=5)	-54.5 ± 3.3 (n=5)		207 ± 39.1 (n=5)	26.9 ± 14.5 (n=5)
**Evoked APs**							
**CNTR** *Working-like*	139.8 ± 68.9* (n=37)	25.3 ± 9.0** (n=37)	107.8 ± 9.7** (n=37)	-62.0 ± 7.4 (n=38)	-82.5 ± 2.5 (n=37)	236 ± 131 (n=37)	18.2 ± 3.4** (n=38)
**LMNA-K219T** *Working-like*	100.0 ± 58.3 (n=35)	18.3 ± 9.2 (n=35)	101.0 ± 9.1 (n=35)	-58.1 ±9.4 (n=35)	-82.8 ± 2.3 (n=35)	184 ± 90.7 (n=35)	22.1 ± 6.3 (n=36)

Supplementary Table 3 - Characterization of spontaneous and evoked action potentials in working-like and pacemaker-like iPSC-derived CMs. dV/dt_{max}, maximal upstroke velocity; Overshoot, peak voltage; APA, action potential amplitude; MDP, maximal diastolic potential; RMP, resting membrane potential induced by current input; APD_{90}, action potential duration measured at 90% repolarization; C_m, membrane capacity. *$p<0.05$, **$p<0.005$ vs LMNA-K219T *working-like* (Student's t-test for unpaired samples).

	**Peak current** (pA/pF)	$V_{1/2}$ **act** (mV)	**k act**	$V_{1/2}$ **inact** (mV)	**k inact**	V_{rev} (mV)	C_m (pF)
**CNTR**	178.7 ± 27.2*** (n=23)	-33.60 ± 3.02 (n=23)	5.36 ± 1.60 (n=23)	-74.78 ± 6.26 (n=18)	6.45 ± 0.80 (n=18)	49.87 ± 7.78 (n=23)	17.40 ± 5.61 (n=23)
**LMNA-K219T**	108.3 ± 26.2 (n=22)	-33.44 ± 4.17 (n=22)	5.57 ± 1.34 (n=22)	-73.47 ± 6.85 (n=17)	6.55 ± 0.76 (n=17)	49.36 ± 6.99 (n=22)	19.45 ± 4.48 (n=22)
**LMNA-R190W**	122.3 ± 37.6 (n=18)	-34.75 ± 5.86 (n=18)	5.83 ± 1.56 (n=18)	-76.61 ± 6.75 (n=14)	6.84 ± 0.54 (n=14)	48.83 ± 8.52 (n=18)	20.42 ± 4.79 (n=18)
**K219T-CORRECTED**	210.8 ± 62.4*** (n=14)	-33.15 ± 3.10 (n=14)	5.14 ± 1.40 (n=14)	-73.25 ± 4.63 (n=12)	6.42 ± 0.85 (n=12)	48.92 ± 6.09 (n=14)	16.34 ± 3.15* (n=14)
**RUES2-GFP^{neg}**	180.8 ± 36.2$^{\&\&}$ (n=14)	-36.18 ± 7.50 (n=14)	4.70 ± 2.79 (n=14)	-69.26 ± 8.27 (n=11)	6.49 ± 2.33 (n=11)	50.42 ± 9.16 (n=14)	18.23 ± 4.94 (n=14)
**LMNA-K219T-GFP^{pos}**	125.4 ± 38.9 (n=21)	-34.40 ± 5.89 (n=21)	5.19 ± 2.10 (n=21)	-72.71 ± 7.91 (n=17)	6.31 ± 1.20 (n=17)	48.93 ± 8.43 (n=21)	20.77 ± 4.78 (n=21)
**LMNA-WT GFP**	192.7 ± 62.3$^{\&\&}$ (n=14)	-33.45 ± 4.28 (n=14)	4.68 ± 1.11 (n=14)	-74.63 ± 5.88 (n=12)	6.47 ± 0.67 (n=12)	48.30 ± 7.36 (n=14)	20.85 ± 4.60 (n=14)
**GFP empty**	196.0 ± 55.0$^{\&\&\&}$ (n=15)	-33.30 ± 2.91 (n=15)	4.80 ± 1.22 (n=15)	-73.92 ± 6.67 (n=12)	6.30 ± 0.82 (n=12)	48.93 ± 8.43 (n=15)	18.37 ± 4.59 (n=15)
**K219T-SCN5A GFP^{pos}**	357.0 ± 90.4$^{\#\#\#}$ (n=14)	-35.75 ± 4.29 (n=14)	3.71 ± 0.87$^{\#}$ (n=14)	-70.16 ± 7.21 (n=12)	5.94 ± 0.65 (n=12)	50.94 ± 5.49 (n=14)	21.28 ± 4.19 (n=14)
**K219T-GFP^{neg}**	124.6 ± 53.0 (n=6)	-33.32 ± 3.61 (n=6)	5.14 ± 1.06 (n=6)	-73.00 ± 8.58 (n=5)	6.24 ± 0.37 (n=5)	46.65 ± 5.23 (n=6)	21.60 ± 3.19 (n=6)
**R190W-SCN5A GFP^{pos}**	338.8 ± 87.3$^{\Theta\Theta\Theta}$ (n=13)	-35.61 ± 3.84 (n=13)	4.18 ± 1.04 (n=13)	-69.23 ± 7.54 (n=11)	5.64 ± 1.34 (n=11)	48.73 ± 6.03 (n=13)	21.04 ± 6.52 (n=13)
**R190W-GFP^{neg}**	111.4 ± 34.9 (n=6)	-33.02 ± 7.07 (n=6)	4.68 ± 1.10 (n=6)	-75.36 ± 6.50 (n=5)	6.50 ± 1.13 (n=5)	50.25 ± 5.75 (n=6)	19.70 ± 4.26 (n=6)

Supplementary Table 4 - Characterization of sodium currents during rectangular double-pulse protocols measured in iPSC-derived CMs. Peak current, average peak I_{Na} density obtained from *I*-

V relations; $V_{1/2}$ act, potential of half maximal activation; k act, slope factor of activation curve; $V_{1/2}$ inact, potential of half maximal inactivation; k inact, slope factor of inactivation curve; V_{rev}, reversal potential; C_m, membrane capacity. * P<0.05; *** P<0.0005 vs LMNA-K1219T and LMNA-R190W; &&<0.005; &&&<0.0005 vs LMNA-K219T-GFP^{pos}; #<0.05, ##<0.005 vs K219T-GFP^{neg}; □□□<0.0005 vs R190W- GFP^{neg} (Student's t-test for unpaired samples).

	dV/dt_{max} (V s^{-1})	Overshoot (mV)	APA (mV)	MDP (mV)	RMP (mV)	APD_{90} (msec)	C_m (pF)
**Spontaneous APs**							
**LMNA-K219T**	11.8 ± 9.2** (n=24)	12.3 ±11.6* (n=24)	74.2 ± 16.1* (n=24)	-61.9 ± 7.0 (n=24)		285 ± 122 (n=24)	21.6 ± 7.7 (n=24)
**K219T-SCN5A GFP^{pos}**	30.0 ± 17.7 (n=18)	21.7 ± 10.4 (n=18)	85.7 ± 13.9 (n=18)	-64.1 ± 7.4 (n=18)		260 ± 106 (n=18)	21.2 ± 4.9 (n=18)
**Evoked APs**							
**LMNA-K219T**	85.6 ± 60.1** (n=46)	14.9 ± 11.1** (n=46)	97.3 ±11.5** (n=46)	-56.8 ± 9.6 (n=46)	-82.4 ± 2.4 (n=46)	165 ± 85 (n=46)	22.0 ± 6.3 (n=46)
**K219T-SCN5A GFP^{pos}**	210.5 ± 77.9 (n=25)	33.9 ± 6.9 (n=25)	117.0 ± 6.5 (n=25)	-57.9 ± 10.8 (n=25)	-83.0 ± 2.2 (n=25)	124 ± 83 (n=25)	21.7 ± 4.4 (n=25)

Supplementary Table 5 - Characterization of spontaneous and evoked action potentials measured in iPSC-derived CMs in which *SCN5A* is overexpressed. dV/dt_{max}, maximal upstroke velocity; Overshoot, peak voltage; APA, action potential amplitude; MDP, maximal diastolic potential; RMP, resting membrane potential induced by hyperpolarizing current input; APD_{90}, action potential duration measured at 90% repolarization; C_m, membrane capacity. * $p<0.05$, ** $p<0.005$ vs K219T-SCN5A GFP^{pos} (Student's t-test for unpaired samples).

**Primers used for ChIP experiments**		
ATCN2	F: 5' GGGCAAGATAGATTTCCCTTCT 3'	R: 5' AGGAGTTTGGGATGTGTGTTCT 3'
CX40	F: 5' ACAATGACTAAGAGCCCACAGAT 3'	R: 5' CTGCATAAAAGTGGGGTAAACTC 3'
CX43	F: 5' CAAACAGCAGCGGAGTTTTA 3'	R: 5' TGAAGTCACGCCAAGTGATT 3'
DEFA 3	F: 5' AACCTGAGGCAAAGATGGTG 3'	R: 5' CTGTCCTTCCAGCCTCTCAC 3'
DEFA 4	F: 5' TGCAGCGAGATTGGTGTTAG 3'	R: 5' AATAACCCAGGAGGGACCAC 3'
JUN	F: 5' ACTCTGAGCCCTTATCCAGC 3'	R: 5' AAAGAAGGGCCCGACTGTAG 3'
NANOG	F: 5' TTGTGGGCCTGAAGAAAACTAT 3'	R: 5' TCAAGAGGGAAATGATCCCTTA 3'
NEUROD	F: 5' GGGGTACTTGAGTGACACGACT 3'	R: 5' TGCCCCAGAATAGAAGCAATAA 3'
SCN5A	F: 5' TGCACACCCTTCCAATAACC 3'	R: 5' GGACAGTGGCAAATTGCAG 3'
SCN10A	F: 5' GGAAGAGGGTGAGGGAAAGC 3'	R: 5' TGTCACCAGTCCTTCCATGC 3'
TNNC1	F: 5' GGAGACAGTAATGGGGACTCAG 3'	R: 5' GGACAGTGATTCTGGTGTTTGA 3'
TBX3	F: 5' GCTTGTGAAACTGATCCCAAAGT 3'	R: 5' GGCCTTTGAAGACCATGGAG 3'
TBX5	F: 5' CGTTCATGAGAAGTAGCAGCAC 3'	R: 5' GGGGGAAATAGGTTTCTCTTTG 3'
**Primers used for gene expression experiments**		
SCN5A	F: 5' GAGCTCTGTCACGATTTGAGG 3'	R: 5' GAAGATGAGGCAGACGAGGA 3'
CX40	F: 5' GGCTCACTGTCCTCTTCATATTC 3'	R: 5' CACACATAGGTGTTGAGCAGAGT 3'
CX43	F: 5' TGAGCGGGTGGTAATTGTGG 3'	R: 5' TGGAGGGTCAGGCCTAGAAA 3'
TBX3	F: 5' AGCAGCTTTCAACTGCTTCG 3'	R: 5' CCATGCTCCTCTTTGCTCTC 3'
TBX5	F: 5' GCATGGAGGGAATCAAAGTG 3'	R: 5' CGTCGGCAGGTACAATGTC 3'
HGPRT	F: 5' GACCAGTCAACAGGGGACAT 3'	R: 5' CTGCATTGTTTTGCCAGTGT 3'
18S	F: 5' CGCAGCTAGGAATAATGGAATAGG 3'	R: 5' CATGGCCTCAGTTCCGAAA 3'
DESMIN	F: 5' GTGAAGATGGCCCTGGATGT 3'	R: 5' TGGTTTCTCGGAAGTTGAGG 3'
DNMT3B	F: 5' ATAAGTCGAAGGTGCGTCGT 3'	R: 5' GGCAACATCTGAAGCCATTT 3'
KRT 14	F: 5' CACCTCTCCTCCTCCCAGTT 3'	R: 5' ATGACCTTGGTGCGGATTT 3'
NCAM	F: 5' CAGATGGGAGAGGATGGAAA 3'	R: 5' CAGACGGGAGCCTGATCTCT 3'

OCT4	F: 5’ GAGAAGGAGAAGCTGGAGCA 3’	R: 5’ AATAGAACCCCCAGGGTGAG 3’
REX1	F: 5’ TCGCTGAGCTGAAACAAATG 3’	R: 5’ CCCTTCTTGAAGGTTTACAC 3’
SOX17	F:5’ GGCGCAGCAGAATCCAGA 3’	R: 5’ CCACGACTTGCCCAGCAT 3’

Supplementary Table 6 - Primes sequences used in the gene expression and ChIP experiments

PCR	Primer	Sequence	Location	Band WT (bp)	Band TARGET (bp)	Band PLASMID (bp)
LOCUS /LoA	LMNA_FW1	CCCCTGCTCAAACATCCTCA	Left to 5' HAR	2181	5260 or LoA	-
	LMNA_REV1	TGCAATCAGAGCTTCCCCAG	Right to 3' HAR			
5'-INT	LMNA_FW1	CCCCTGCTCAAACATCCTCA	Left to 5' HAR	-	1154	-
	PB3-P2	GCGACGGATTCGCGCTATTTAGAAAG	5' ITR			
5'-INT+ 5'-LOCUS	LMNA_FW1	CCCCTGCTCAAACATCCTCA	Left to 5' HAR	1836	1154 (4915)	-
	PB3-P2	GCGACGGATTCGCGCTATTTAGAAAG	5' ITR			
	LMNA_REV2	CTGTGGTTGTGGGGACACTT	3' HAR			
3'-INT	PB5-P2	CGTCAATTTTACGCATGATTATCTTTAAC	3' ITR	-	1173	-
	LMNA_REV1	TGCAATCAGAGCTTCCCCAG	Right to 3' HAR			
3'-INT + 3'-LOCUS	LMNA_FW2	CCCAAAAGTACCCCAGGCAT	5' HAR	1900	1173 (4979)	-
	PB5-P2	CGTCAATTTTACGCATGATTATCTTTAAC	3' ITR			
	LMNA_REV1	TGCAATCAGAGCTTCCCCAG	Right to 3' HAR			
5'-BB	M13-R49	GAGCGGATAACAATTTCACACAGG	Plasmid 5'	-	-	1157
	PB3-P2	GCGACGGATTCGCGCTATTTAGAAAG	5' ITR			
3'-BB	PB5-P2	CGTCAATTTTACGCATGATTATCTTTAAC	3' ITR	-	-	1127
	M13-F43	AGGGTTTTCCCAGTCACGACGTT	Plasmid 3'			

Supplementary Table 7 - Genotyping strategies for the characterization of CRISPR/Cas9-edited human iPSCs.

Extended Methods

Patients' clinical information. iPSC lines were generated from fibroblasts isolated from skin biopsies of 3 members of a family carrying the K219T mutation (referred to as LMNA #1, LMNA #2 and LMNA #3) and diagnosed with dilated cardiomyopathy (DCM) [1]. iPSCs were also generated from two healthy wild-type members and used as family-matched controls. Detailed information on patients and control subjects are available in Supplementary Table 1 and representative electrocardiogram (ECG) traces shown in Supplementary Figure 1.

In particular, we collected clinical data regarding conduction abnormalities, namely ECG traces, electrophysiological study (EPS), echocardiography and relevant clinical events, such as pacemaker (PM) and implantable cardioverter defibrillator (ICD) implantation and history of onset of spontaneous arrhythmias. Relevant arrhythmic events were recorded in patients LMNA #2 and LMNA #3. Patient LMNA #2 presented with left posterior fascicular block and second-degree atrioventricular block, and underwent PM implantation in 2000; the patient was upgraded to biventricular ICD in 2003. The EPS was negative for inducible arrhythmias, and patient history was negative for spontaneous arrhythmias, except for episodes of paroxysmal atrial fibrillation before transplant, in 2005. Patient LMNA #3 developed atrial fibrillation, left bundle branch and a third-degree atrioventricular block, and underwent PM implantation in 2000, and was upgraded to biventricular ICD in 2005. Despite having a negative EPS for inducible ventricular arrhythmias, the patient had episodes of ventricular tachycardia and ventricular flutter before heart transplant, in 2006. Patient LMNA #1 was diagnosed with initial DCM with no signs of conduction abnormalities: in fact, the ECG showed no alterations of heart rhythm, with normal atrioventricular and interventricular conduction, and echocardiography study did not show interventricular septal motion abnormalities. The latter, present in other members of the family not included in the present study, was an early sign of delayed conduction before the manifestation on the ECG.

An additional case carrying the *p.*R190W *LMNA* mutation was also enrolled in the study and iPSCs generated from peripheral blood mononuclear cells. The patient, a male that was 39 years old

at the time of enrolment, was diagnosed with DCM and presented with frequent ventricular ectopic beats.

iPSC generation, characterization and maintenance. Reprogramming was achieved by transduction of the four "Yamanaka" factors using the STEMCCA lentiviral vector[2], kindly provided by Dr. Mostoslavsky (Boston University School of Medicine, MA, USA).

Characterization entailed analyses of expression of pluripotency markers (Oct4, Rex1, Dnmt3B, Tra1-60, SSEA4) either by real time PCR or immunofluorescence, embryoid bodies, and teratoma assays to assess the ability of reprogrammed cells to differentiate into derivatives of the three germ layers *in vitro* and *in vivo*[3]. Analysis of the karyotype was also carried out by standard procedures[4]. Two clones per patient were used for the experiments.

Cells were maintained under 5% CO_2 in Essential 8 medium on Vitronectin (both from ThermoFisher Scientific) and passaged 1:5 when 90% confluency was reached (~4-5 days), using 0.5mM EDTA after pre-treatment with 10 µM Rho-associated protein kinase inhibitor (Rocki from Selleck Chemicals).

All cell lines were negative for mycoplasma contamination.

Cardiac differentiation. iPSCs were differentiated into iPSC-CMs using a monolayer differentiation protocol[5], and were maintained in a 5% CO_2/air environment. In brief, iPSC colonies were dissociated with Accutase (Gibco-Life Technologies) and plated at a density of 0.25–0.5 x 10^5 cm^{-2} in 12-well plates coated with Growth Factor Reduced (GFR) Matrigel (BD Bioscience) in Essential 8 medium containing 10 µM Rocki. Cells were cultured until they reached 95–100% confluence (~4 days) and then treated for 24 hours with 8–12µM CHIR99021 (Selleck Chemicals) in RPMI supplemented with B27 without insulin (the day of the treatment is referred to as day 0). On day 3, cells were treated with 5µM IWR-1 (Sigma-Aldrich) for 48 hours. Medium was replaced on days 5 and 7 with RPMI+B27 without insulin; from day 10, RPMI supplemented with B27 and insulin was used and changed every other day. Cells were used for the experiments 20–25 days after the start of spontaneous contractions.

Data presented are average of values obtained in CMs differentiated from two iPSCs lines per subject (2 CNTR and 3 K219T-LMNA).

FACS analysis. Differentiation efficiency of iPSC lines was evaluated by FACS through the assessment of expression of the cardiac-specific marker α-sarcomeric actinin[6]: CMs were enzymatically dissociated into single cells, fixed in 1% paraformaldehyde and permeabilized; staining and detection were then performed using mouse monoclonal α-sarcomeric actinin antibody (1:400 from Abcam, Cambridge, UK) and goat anti-mouse Alexa-647-conjugated antibody (1:500 from Molecular Probes, Thermo Scientific), respectively.

Dead cells were quantified and excluded from the analysis using the LIVE/DEAD fixable aqua stain kit (Molecular Probes, Thermo Fisher Scientific). Cell acquisition was performed on a FACS LSRFortessa flow cytometer (BD Bioscience, San Jose, CA, USA) and data analysed using DIVA software (BD Pharmingen, San Diego, CA, USA).

Patch-clamp recordings. Electrophysiological recordings were performed using a Multiclamp 700B patch-clamp amplifier (Molecular Devices) controlled by pClamp 10.3 software (Molecular Devices). Low-density cell cultures grown on glass coverslips (VWR International) were transferred 2–3 days after seeding to a custom-made experimental chamber that was fixed to the stage of an inverted microscope (Nikon eclipse, *T*i/U) and superfused at 1.5 mL/min. Single human iPSC-derived cardiomyocytes (iPSC-CMs), identified on the basis of their typical morphology, were current/voltage-clamped in the whole-cell configuration of the ruptured patch-clamp technique. Data were collected from a minimum of three independent differentiations per line. Na^+ current (I_{Na}) and action potential (AP) signals were low-pass filtered at 10 kHz, digitized at 20 and 50 kHz, respectively, and stored for offline analysis whereupon the latter was performed using dedicated software (pClamp 10.6 and Clampfit 10.6; Molecular Devices). Patch pipettes were pulled from borosilicate glass capillaries (Intrafil-10, INTRACEL LTD) with a laser-based micropipette puller (P-2000; Sutter Instrument) and resistances ranging from 1 to 3 MΩ. After seal formation (2-10 G), rupturing of the patch, and before I_{Na} measurement, adequate voltage control was achieved with membrane capacitance (C_m) and series resistance (R_s: 3–8 MΩ) compensation above 80% (estimated

voltage error <5 mV in all cases), and by maintaining a fraction of channels in their inactivated state by using a holding potential of -90mV. C_m was determined by dividing the decay time constant of the whole-cell capacitance transient in response to hyperpolarizing steps from -50 mV, by the R_s. Statistical comparisons were made using Student's t-test for unpaired samples. Differences with at least $p<0.05$ were regarded as significant.

AP measurements*:* AP characteristics were assessed at 36°C using Hanks' balanced salt solution (HBSS, Sigma-Aldrich) containing (in mmol L^{-1}): NaCl 137, KCl 5.4, $CaCl_2$ 1.3, $MgSO_4$ 1.2, $NaHCO_3$ 4.0, KH_2PO_4 0.5, NaH_2PO_4 0.3, D-glucose 5.5 and HEPES 10 (pH 7.40 with KOH). The pipette filling solution contained (in mmol L^{-1}): K-aspartate 120, NaCl 10, MgATP 3, $CaCl_2$ 1, EGTA 10, HEPES 5 (pH 7.2 with KOH). Liquid junction potential corrections (12.4 mV as calculated by pCLAMP software; Axon Instruments) were applied after experiments during offline analysis. AP measurement experiments were conducted on either spontaneously active iPSC-CMs or iPSC-CMs in which the MDP was adjusted to physiological resting cardiac membrane potentials (i.e. ~ -80 mV) by electrical injection of a constant hyperpolarizing current. Maximal upstroke velocity (dV/dt_{max}), peak voltage (overshoot), action potential amplitude (APA), action potential duration at 90% of repolarization (APD_{90}), and maximal diastolic potential (MDP) were analysed. APs were recorded from cells able to generate spontaneous APs, averaging the 1/2 last sequential APs obtained from 5/10 sec current-clamp recordings. APs obtained from cells electrically maintained at ~ -82 mV (i.e. resting membrane potential (RMP) range from -77.4 to -87.2mV) were evoked through depolarizing current injections just above the threshold intensity to guarantee that the maximal upstroke velocity was not affected by the stimulation artefacts (1.2 x threshold; duration: 1.5 ms; amplitude: 300–800 pA; cycle time between current steps: 2 sec). The hyperpolarizing current input used to generate RMPs was 3.0±1.2* pA/pF (n=45) for CNTR-CMs and 3.6±1.7 pA/pF (n=46) for LMNA-CMs; $p<0.05$.

***I_{Na}* measurements:** Whole cell I_{Na} recordings were conducted at room temperature in an extracellular solution containing (in mmol L^{-1}): NaCl 70, $CaCl_2$ 1.8, CsCl 90, $MgCl_2$ 1.2, glucose 10, HEPES 10, and Nifedipine 0.001 (pH 7.4 with CsOH) and pipette solution filled with (in mmol L^{-1}): CsCl 130,

NaCl 10, $CaCl_2$ 1, EGTA 10, HEPES 5, Mg-ATP 3 (pH 7.2 with CsOH). Potentials were corrected for the estimated change in liquid junction potential of 6 mV before experiments. Peak I_{Na} densities and the voltage-dependence of activation were characterized as follows: single cells were held at -90mV and 100 msec voltage steps were applied from -90 to +80mV in 5 mV increments with a cycle time between voltage steps of 3 sec. Voltage-dependence of inactivation was assessed by holding cells at various potentials from -130 to 15 mV followed by a 50 msec test pulse to -20mV to elicit I_{Na} (holding potential of -90 mV). To take into account the variations in cell size, current amplitude was normalized to C_m and expressed as current density (pA/pF). Activation and inactivation curves were described by fitting experimental points with the Boltzmann equation to estimate the voltages of half-maximal activation and inactivation ($V_{1/2}$) and each slope-1 factor (k).

Assessment of MDP and dV/dt$_{max}$ impact on I_{Na} derived from different populations of LMNA-CM AP phenotypes: further electrophysiological considerations: In this study, we used CMs generated from iPSCs to investigate two electrophysiological parameters which are critical for cardiac cellular excitability and arrhythmogenesis: the maximal upstroke velocity of the AP (dV/dt$_{max}$) and maximal diastolic potential (MDP). In mature atrial and ventricular CMs, the dV/dt$_{max}$ can be directly translated into the peak Na^+ current (I_{Na}) that is activated through fast voltage-dependent Na^+ channels (Na_v1.5) during the AP upstroke, while MDP represents the main control for the activity of Na_v1.5. When MDP is shifted in depolarizing direction, Na_v1.5 will be mostly inactivated and disabled to concur to the AP upstroke[7,8]. Since iPSC-derived CMs may present heterogeneity in ion channels repertoire (e.g., profound variabilities in I_{Na} and K^+ currents, which stabilize the resting membrane potential of iPSCs, have been reported [9,10]), in this work we took in consideration the different CMs subpopulations (typified by specific AP characteristics) and analysed how these affected the measure of I_{Na}.

AP electrophysiological properties were obtained from the following CNTR- and K219T-CM subpopulations: spontaneously beating iPSC-CMs; iPSC-CMs in which the MDP was adjusted to physiological resting cardiac membrane potentials (i.e., ~ -80 mV) by electrical injection of a constant hyperpolarizing current; working-like spontaneously beating iPSC-CMs; and working-like iPSC-

CMs electrically forced to ~ -82 mV. As expected, MDPs did not significantly change in the spontaneously beating CNTR- and K219T-CMs (-63.9±5.6 mV, n=33 VS. -61.9±7.0 mV, n=24; see Fig. 1B and Supplementary Table 2) in comparison to MDPs obtained from either quiescent or beating iPSC-CMs (Fig. 1B and Supplementary Table 2). This finding is explained by mechanisms of depolarization-induced automaticity[11,12], where appearance of spontaneous activity is a function of MDP and single beating cells are generally characterized by less-negative MDPs. Based on the same principle, a higher degree of membrane depolarization, as measured in K219T-CMs, invariably stopped spontaneous activity in a higher number of mutant cells than controls (48% vs 28%). Nonetheless, MDPs obtained from spontaneously beating iPSC-CMs were in a voltage range where any change in MDP plays an important effect on Na^+ inactivation[13]. Based on this consideration, the presence of a few mVs of membrane potential difference between spontaneously beating CNTR- and K219T-CMs cannot be considered negligible because it might have an important impact on $Na_V1.5$ availability and, as a consequence, on dV/dt_{max} size[14]. The latter was bypassed by electrically adjusting the MDP of CNTR- and K219T-CMs to ~ -82mV in order to obtain the same levels of $Na_V1.5$ de-inactivation and further characterize dV/dt_{max} at more physiological resting cardiac membrane potential[15]. In this condition, the 36.1% dV/dt_{max} reduction measured in K219T-CMs vs CNTR-CMs (Supplementary Figure 6) resulted decreased to 31.4% (4.7% less) in K219T-CMs electrically adjusted to ~ -82mV (Supplementary Figure 6). The 4.7% discrepancy in the AP upstroke velocity between the two experimental settings might be a consequence of the slightly more depolarized membrane potential of spontaneously beating K219T-CMs vs CNTR-CMs and, therefore, the 31.4% I_{Na} decrement is the real outcome of the reduced $Na_V1.5$ expression in CMs carrying the K219T Lamin A/C mutation.

Another well-described issue faced when approaching electrophysiological studies of CMs obtained from pluripotent stem cells is the heterogeneity of AP morphologies of the generated cells (typically a mixed population of atrial-like, ventricular-like and pacemaker-like cells typified by different AP parameters, with most of the cells possessing a ventricular phenotype)[9,16,17]. Therefore, in the context of MDP and dV/dt_{max} characterization of iPSC-CMs with different AP profiles, we

selected CMs with atrial/ventricular characteristics, named here as working-like CMs, based on their AP properties using criteria we had previously determined[3]. This additional analysis was performed to avoid possible I_{Na} underestimations due to contamination with pacemaker cells, which are more depolarized and devoid of Na_V1.5 (indeed, they displayed calcium-based activity only). As expected, no significant differences between AP parameters of pacemaker-like CNTR- and K219T-CMs were measured (Supplementary Table 3). Importantly, working-like spontaneous AP parameters recorded in K219T-CMs were significantly decreased with respect to CNTR-CMs (Supplementary Table 3), with a decrement in dV/dt_{max} of 29.8% (Supplementary Figure 6). There are two important factors to consider when explaining the 6.3% discrepancy in the reduction of the dV/dt_{max} between the spontaneously beating iPSC-CMs and the respective working-like iPSC-CM population. First, there was greater contamination with pacemaker-like APs in iPSC-CM preparations derived from mutant K219T-iPSC lines with respect to those generated for the CNTR lines (5 out of 24 vs 3 out of 33). Furthermore, two cells per condition showed AP profiles which were non-classifiable as pacemaker- or working-like. Second, working-like spontaneously beating CNTR- and K219T-CMs had very similar MDPs, with an MDP difference reduced to 0.2 mV with respect to that between all spontaneously beating CNTR- and K219T-CMs (i.e., 2 mV). Noteworthy, dV/dt_{max} decrements such as those registered in both mutant CM populations, working-like spontaneously beating iPSC-CMs and those electrically adjusted to ~ -82 mV, were also very similar (i.e., 29.8 and 31.4%). This finding can be explained as above. In fact, when the dV/dt_{max} analysis was restricted to working-like CNTR- and K219T-CMs electrically adjusted to ~ -82 mV, a 28.4% I_{Na} decrement was measured (Supplementary Figure 6). This value was similar to the 29.8% and 31.4% decrements obtained in working-like spontaneously beating CMs and in the iPSC-CMs electrically adjusted to ~ -82 mV, respectively. Indeed, the MDP difference between working-like, spontaneously beating CNTR- and K219T-CMs (only 0.2 mV) virtually matched the MDP difference between CMs electrically adjusted to ~ -82 mV (i.e., 0.0 mV in CNTR- and K219T-CMs electrically adjusted to ~ -82 mV, and 0.3 mV in working-like CNTR- and K219T-CMs electrically adjusted to ~ -82 mV).

In conclusion, the decreased cellular excitability measured in mutant CMs with respect to the controls is coherent with both the different AP characteristics and the biophysical parameters of the analysed iPSC-CM subpopulations: this validates the use of our LMNA-CMP model for the characterization of I_{Na} and conduction activity in heart muscle.

Optical measurements of impulse conduction: Action potential propagation along 80µm-wide strand preparations was assessed optically following staining for 4 min with the voltage-sensitive dye di-8-ANEPPS (135 µmol L^{-1}; Life Technology)[8]. Stained preparations were mounted in a temperature-controlled chamber (University of Bern, CH), placed on the stage of an inverted microscope (Nikon Ti/U) and superfused at 36°C with HBSS containing 1% FBS. Strands were either left to beat spontaneously or stimulated with a bipolar electrode placed ≥1 mm from the measurement site in order to permit AP propagation to reach steady-state conditions at the recording site. Strands were stimulated for the entire duration of recording (4 seconds, 5 stimuli) with a stimulator (TTi 110, Harward Apparatus) at a basic cycle length of 500 ms or 1000 ms. Recordings were made at 10x magnification (S-Fluo 10x, 0.5 N.A., Nikon), which allowed us to monitor the activity of two strands in one field of view. Impulse propagation was acquired with a fast-resolution camera at 1–5 kHz temporal resolution (Ultima L, Scimedia, USA). The amplitude of optically recorded AP upstrokes was scaled to 10% (dF/F). Conduction velocities were computed by calculating the activation times for a given strand and dividing by the covered space of the propagation, with dedicated software (BrainVision AN 1812).

To assesses the ionic driver of optical impulse propagation, we added 100 mol/l of tetrodotoxin (Alomone Lab) to the superfused HBSS solution, and recorded conduction velocities after 10 minutes.

Lentiviral overexpression. For overexpression experiments, a lentiviral construct encoding C-terminus GFP-tagged Lamin A/C carrying the K219T mutation (c. A656C) was obtained from Origene in the pLenti-EF1alpha-GFP vector (CW104035). We also generated a lentiviral construct encoding C-terminus GFP-tagged wild-type Lamin A/C by subcloning the WT *LMNA* gene into the same pLenti-EF1alpha-GFP vector backbone. An additional pLenti-EF1alpha-SCN5A-GFP,

encoding C-terminus GFP-tagged *SCN5A*, was also obtained from OriGene (CW104036). Lentiviral particles were produced in HEK293T cells and used to transduce iPSC-derived CMs. For Lamin A/C overexpression experiments, transduction was performed between days 5 and 6 of the differentiation protocol, a stage at which Lamin A/C starts to be expressed in this system; cells were used for electrophysiology and gene expression studies 20 days after the start of spontaneous beating. For gene expression, cells were sorted for GFP expression with a BD FACS ARIA III cell sorter and harvested for RNA extraction.

Overexpression of the *SCN5A* gene was obtained by transduction of lentiviral particles in mutant iPSC-CMs at the end of differentiation (after contraction started).

Generation of isogenic control iPSC lines. To allow "scarless" editing, we identified a suitable endogenous "TTAA" site in the third intron of *LMNA* and located 135 bp upstream of the AC mutation in exon 4 leading to the *p.*K219T change (Supplementary Fig. 14A). Further, we designed a single guide RNA (sgRNA) spanning the TTAA site, so that only the wild-type sequence could be cut by CRISPR/Cas9. This strategy allowed us to avoid inserting any additional mutation into the targeting vector homology arms (such as those classically used to disrupt the PAM site), so preventing any unforeseen interference with the regulatory elements of the *LMNA* gene. The sgRNA was designed using mit.crispr.edu and had a score of 80%, indicating a very high in-silico predicted specificity (sequence without PAM site: 5′-CTACCAGCCCCACTTTAACC-3′). To further decrease the risk of CRISPR/Cas9 off-target activity, we adopted enhanced specificity SpCas9 (eSpCas9), developed by Dr. Feng Zhang and colleagues[18]. The sgRNA was cloned into the eSpCas9(1.1) plasmid (Addgene #71784) using a standard method based on restriction digestion with BbsI followed by ligation of a double-stranded oligonucleotide[19]. The resulting plasmid was named eSpCas9(1.1) LMNA. The sequence was confirmed by Sanger sequencing, and the sgRNA was validated to have a high on-target activity, as measured by T7E1 assay in HEK293 cells (which was comparable to that observed using conventional SpCas9).

The *LMNA* targeting vector was constructed starting from the MV-PGK-Puro-TK_SGK-005 plasmid (Transposagen), which contains a piggyBac transposon encoding a PGK-EM7 promoter-

driven dual positive/negative selection cassette (puromycin N-acetyltransferase, ensuring resistance to puromycin, and truncated thymidine kinase, conferring sensitivity to ganciclovir or its analogue fialuridine). First, the piggyBac cassette was excised using NsiI and BsiWI and isolated. Then, a backbone with ends suitable for the subsequent overlap-based assembly was obtained from this same plasmid after removal of the piggyBac cassette using NotI and AscI. Finally, these two fragments were re-assembled together with two PCR products representing the 5′ and 3′ homology arms to the *LMNA* gene. The two homology arms were approximately 1 Kb long, and were amplified from genomic DNA of RUES2 human embryonic stem cells (ESCs) using primers: 5′-GGTCCCGGCATCCGATACCCAATGGCGCGCCCGTACTTCAGGCTTCAGCAGT-3′ and 5′-AAAGAGAGAGCAATATTTCAAGAATGCATGCGTCAATTTTACGCAGACTATCTTTCTA GGGTTAACCTGGGAGCTGAGTGC-3′ (for the 5′ homology arm); 5′-AATTTTACGCAT GATTATCTTTAACGTACGTCACAATATGATTATCTTTCTAGGGTTAAAGTGGGGCTGGT AGTG-3′ and 5′-CGAATGCGTCGAGATATTGGGTCGCGGCCGCCCTGTCACAAATAGCA CAGCC-3′ (for the 3′ homology arm). The four-way assembly reaction was performed using NEBuilder HiFi DNA Assembly Kit (New England Biolabs), according to the manufacturer's instructions, and the resulting targeting plasmid was named pbLMNA_K219K. Sanger sequencing confirmed that the 3′ homology arm contained the wild-type K219K allele, while the remaining genomic sequence of both homology arms was identical to that of line LMNA #1_2 as no SNPs were identified. The cloning strategy was designed so that during PCR the "TTAA" site was inserted both at the end of the 5′ homology arm and at the start of the 3′ homology arm, ensuring that the piggyBac cassette contained within could be excised using transposase while leaving behind a single "TTAA" matching the original genomic sequence (Fig. 9A and Supplementary Fig. 14A).

For the first gene targeting step, 150,000 hiPSCs (line LMNA #1_2) were seeded in each well of 6-well plate and immediately transfected using GeneJuice (Millipore), according to the manufacturer's instructions. Briefly, for each well, 3 μl of GeneJuice was mixed with 100 μl of Opti-MEM (ThermoFisher Scientific) and incubated for 5 min at room temperature. 1 μg of DNA was added to the transfection solution (equally divided between eSpCas9(1.1) LMNA and

pbLMNA_K219K), which was further incubated for 15 min at room temperature and finally added to the cell suspension. After 16 h from transfection, cells were washed with DPBS and cultured for another 3 days in Essential 8 medium. Media without penicillin/streptomycin was used from two days before transfections up to this point, and 10 μM Y-27362 (ROCK inhibitor; SelleckChem) was added during plating and left until the subsequent day to increase cell survival. Gene targeted cells were selected by adding 1 μg/ml puromycin to the media for 4 days, after which the dose was reduced to 0.5 μg/ml. 10 μM Y-27362 was added for the first 48 h of selection. Puromycin was then maintained at all times until the second gene targeting step to prevent silencing of the piggyBac transgene. After ~10 days from transfection, 5–10 individual and well-separated colonies could be identified in each well of the 6-well plate, indicating that they likely arose from clonal expansion of a single gene-edited human iPSC clone. Colonies were manually picked following gentle treatment with EDTA to facilitate their detachment from the matrix, and individually expanded as individual lines. Clones were screened by genomic PCR using LongAmp Taq Polymerase (New England Biolabs), according to manufacturer's instructions, except that all reactions were performed using an annealing temperature of 63°C and an extension time of 2 min. The primer sequences are reported in Supplementary Table 7 and the genotyping strategies are illustrated in Supplementary Figure 14A. Briefly, junctional PCRs for both the 5′ and 3′ integration site (5′- and 3′-INT) were used to confirm site-specific integration, while locus PCRs were used to monitor the presence of residual wild-type alleles. This allowed to discriminate homozygous clones from heterozygous ones or mixed cell populations. Finally, PCRs of the targeting vector backbone (5′- and 3′-BB) were performed to exclude random integration of the plasmid elsewhere in the genome. Homozygous clones with only on-target integration events were selected (3 out of the 12 lines screened). These positive clones were further characterized by Sanger sequencing of the 5′- and 3′-INT PCR product to confirm the presence of the wild-type K219K allele in homozygosity (2 out of 3 lines) and exclude other unwanted mutations elsewhere in the locus (absent in all lines). One clone was karyotyped by standard G-banding, which confirmed its genetic stability, and therefore selected for the second gene targeting step. This clone was named K219K-PB_cl14. To remove the piggyBac and restore the *LMNA* locus

to its original form, K219K-PB_cl14 iPSCs were transfected as described above but using 1 µg of excision-only piggyBac transposase expression vector (PBx; Transposagen). Puromycin was removed from the media the day before transfection, and subsequently omitted. After 3 days from the transfection, the population was passaged as single cells, and 10,000 cells seeded per 10 cm dish in the presence of 10 µM Y-27362. On the next day, negative selection of cells still possessing the piggyBac cassette was initiated by adding 400 nM fialuridine. 10 µM Y-27362 was added for the subsequent 48 h. Selection was complete after 7 days, at which point 10–50 individual and well-separated colonies could be identified in each 10 cm dish. Individual colonies were isolated, clonally expanded and screened by genomic PCR, as described above, to identify those with homozygous reconstitution of the wild-type allele (3 out of 15 screened lines). These were further characterized by sequencing to ensure that the sequence surrounding the "TTAA" site was faithfully reconstituted upon piggyBac excision. The three clones were karyotyped by standard G-banding, which confirmed their genetic stability. Subsequent functional experiments were conducted on two of those (#F4 and #F6), named K219T-corrected.

Gene expression studies: Total RNA was extracted with Trizol and quantified using Nanodrop (NanoDrop 2000c Spectrophotometer Thermo Fisher Scientific). RNA was reverse transcribed with SuperScript VILO cDNA Synthesis Kit (Thermo Fisher Scientific) according to the manufacturer's instructions. Quantitative reverse transcriptase PCR (qRT-PCR) was performed using SYBR Select Master Mix for CFX (Thermo Fisher Scientific). The relative expression was calculated as 2-ΔΔCt using the comparative Ct method and *18S* or *HGPRT* as housekeeping controls. Specific primer sequences are summarized in Supplementary Table 6.

Western blotting. Protein lysates (30µg) from CNTR- and K219T-CMs were loaded on precast polyacrylamide gels (Bolt 4-12% Bis-Tris Plus Gels, Invitrogen) and transferred to nitrocellulose membranes. Membranes were blocked with 5% milk in TBS–0.1% Tween for 1 hour at room temperature and then incubated overnight with primary antibodies (anti-Na_v1.5 1:1000 from Cell Signaling, D9J7S; anti-β-actin 1:2000 from Santa Cruz, sc1615). Detection was performed after one hour incubation with the appropriate secondary antibody (1:2000) using the Millipore

Chemiluminescent HRP substrate kit. Quantification of $Na_v1.5$ was determined by densitometric analysis relative to β-actin, using Fiji-ImageJ software.

Co-immunoprecipitation (Co-IP) experiments. Co-IP was performed as described by Marullo et al[20]. In brief, nuclear extracts were obtained using PARISTM KIT (Ambion AM1921). 0.5 mg of nuclear extracts were immunoprecipitated overnight at +4° with 5 µg antibodies (Lamin A/C, Santa Cruz mAb sc-7292-x; unrelated IgG) in a nuclei lysis buffer (10 mM Hepes, pH 7.5, 1.5 mM MgCl2, 150 mM KCl, 10% glycerol, 0.2% NP-40, 1 mM DTT, and protease inhibitors). Eluates were analysed by Western blot as described above, using anti-Lamin A/C (Santa Cruz mAb sc-7292-x, 1:2000) and anti-Ezh2 (ACTIVE MOTIF mAb catalogue #39875, AC22 clone, 1:1000) antibodies.

Immunofluorescence. iPSC-CMs were dissociated and plated onto glass cover-slips coated with fibronectin and laminin[5]. Cells were prepared for staining by fixing in 4% paraformaldehyde; cells were then rinsed with PBS–0.1% Tween, blocked with 3% goat serum and permeabilized with 0.1% Triton X-100 for 1 hour at room temperature. Cells were then incubated overnight at +4°C with the primary antibodies: anti-$Na_v1.5$ channel (anti-rabbit from Alomone, #ASC005; 1:100) and anti-α-sarcomeric actinin (Abcam EA-53 ab9465, 1:100). For detection, cells were washed three times with PBS–0.1% Tween and incubated for 1 hour at room temperature with Alexa-Fluor 488- and 555-coniugated secondary antibodies (Molecular Probes from Thermo Scientific, 1:500) raised in the appropriate species for the experiments. Nuclei were stained with DAPI. Images were acquired on an Olympus FV1000 confocal laser-scanning microscope with a x60 oil immersion objective and analysed using ImageJ Fiji software.

For tissue immunofluorescence staining, paraffin-embedded heart sections (3µm thick) from three healthy controls (commercially available from Proteogenex) and four patients were used. Paraffin was removed from tissue sections with sequential treatment with xylene, 100% EtOH, 95% EtOH, and 70% EtOH, and slides subsequently rehydrated with ddH_2O. Prior to immunostaining, we performed antigen retrieval by leaving sections at 98°C for 25 minutes in citrate.

Slides were blocked with 3% goat serum and permeabilized with 0.3% Triton X-100 for 40 minutes at room temperature. Tissue sections were then washed three times with PBS-T (Tween

0.05%) for 5 minutes and then incubated overnight at +4°C with primary antibodies (anti-rabbit $Na_v1.5$, 1:100, from Alomone, #ASC005; anti-mouse α-sarcomeric actinin, 1:100, from Abcam, EA-53 ab9465). After 3 washes with PBS–0.1% Tween, slides were incubated with secondary antibodies for 1 hour at room temperature (Alexa Fluor 555-coniugated goat anti-rabbit, 1:500; Alexa Fluor 488-coniugated goat anti-mouse, 1:500, both from Molecular Probes). Nuclei were detected with DAPI.

Images were acquired at x100 on an Olympus FV-1000 confocal laser-scanning microscope with an oil immersion objective and analysed using ImageJ Fiji software.

Stimulated emission depletion (STED) microscopy. iPSC-CMs were plated on glass coverslips (18mm, #1.5) and prepared for staining by fixing in 2% paraformaldehyde. Cells were then rinsed with PBS–0.1% Tween, blocked with 3% goat serum and permeabilized with 0.1% Triton X-100 for 1 hour at room temperature. Cells were then incubated overnight at +4°C with the primary antibodies: anti- Lamin A/C (anti-mouse, from Santa Cruz 7292-X, 1:250) and anti-Suz12 (anti-rabbit, from Cell Signaling #3737, 1:250). After three washes with PBS–0.1% Tween, cells were incubated for 1 hour at room temperature with Abberior STAR-RED and STAR 580-conjugated secondary antibodies (1:500) raised in the appropriate species for the experiments. Images were acquired on a Leica TCS SP8 STED 3X microscope in "counting mode" with a 100x oil immersion objective, using detection lasers at wavelengths of 634 and 561 nm, and a 775 depletion laser, with a z-step of 60nm. Colocalization analysis was performed with Imaris software (Bitplane). We measured the number of voxels on the 3D reconstructions of Lamin A/C-Suz12 colocalization channel. Statistical analysis was performed using an unpaired Mann-Whitney test with 95% confidence interval.

Distance distribution analysis. PRC2-LaminA/C distribution profile was calculated using the "Distance transformation" Xtension of Imaris Bitplane[21]. The spot option was used to count point-like objects in all acquired z-stacks. Lamin A/C, Suz12 and colocalization channels were re-created using spot settings and visualized as "spheres" with an estimated XY diameter of 0.227 m and a threshold quality above 0.800 fluorescent signal for Lamin A/C; and XY diameter of 0.227 m and a threshold quality above 0.300 fluorescent signal for Suz12 and colocalization channels. The outside "ring of spots" of Lamin A/C was selected and used as the "nucleus edge" to calculate Suz12 and

colocalization channel distances and distributions within nuclei. Data were further analysed by dividing cell nuclei into three zones of 2.15 m in length from periphery to the centre, as schematically represented in Fig. 7D (left panel).

3D-FISH. Immunofluorescence coupled with DNA in situ hybridization on preserved nuclei (3D DNA immuno-FISH) was performed on sterilized 18-mm round coverslips in 12-well tissue culture dishes. The nuclear lamina was visualized using an anti-LMNA antibody (goat IgG, from Santa Cruz Biotechnology, sc-6215 N18). A DNA hybridization probe was generated by nick translation (Nick translation DNA labelling system – Enzo Life Sciences) in the presence of Spectrum Orange dUTP nucleotide (Vysis) of the RP11-356A22 BAC (Children's Hospital, Oakland, CA) containing the *SCN5A* sequence gene.

CNTR-CMs and K219T-CMs were fixed in 2% paraformaldehyde on slides. Cells were permeabilized and incubated for 30 min in blocking solution (3% normal rabbit serum, 0.4% Triton X-100). Cells were then incubated with the primary antibody (1:100) overnight at 4°C. After overnight incubation, samples were washed three times with PBS–Tween 0.1% and incubated with Alexa Fluor 488-coniugated secondary antibodies (Invitrogen, A-11078) diluted 1:500 for 1 h at room temperature. After rinsing, the samples were permeabilized with ice-cold detergent (0.7% Triton-100/0.1M HCl) and FISH was performed to detect SCN5A localization. The labelled probe was resuspended in hybridization buffer (50% formamide, 10% dextran sulphate, 1x Denhart's solution, 0.1% SDS, 40 mM Na_2HPO_4, pH 6.8, 2xSSC) containing a 10x excess of human Cot1 DNA (Life Technologies), and denatured at 80°C for 10 min. In situ hybridization was essentially as previously described[4,22], with minor modifications. In brief, slides were denatured in 70% formamide/2xSSC at 78°C for 10 min, and hybridized for 48h at 37°C. Stringent washings were done with 50% formamide/2xSSC at 37°C. Slides were mounted with Vectashield mounting medium and DAPI, and then scored under an Olympus FV-1000 confocal laser-scanning microscope at x100 with an oil immersion objective, using a x2 optical zoom with a z-step of 0.3 μm between optical slices. Distances between each SCN5A loci and the nuclear periphery were measured with Fiji-ImageJ software through a 3D distance map construction on the DAPI channel.

Chromatin immunoprecipitation. Differentiated iPSC-CMs were fixed in 1% formaldehyde for 10 min and then quenched with 0.125M glycine for 5 min. Fixed cells were lysed in IP buffer made from two volumes of SDS buffer (100mM NaCl, 50mM Tris-HCl pH 8.1, 5mM EDTA pH 8.0, 0.5% SDS), one volume of Triton buffer (100mM NaCl, 100mM Tris-HCl pH 8.1, 5mM EDTA pH 8, 5% Triton X-100) and protease inhibitors (Roche) for 10 min on ice. Chromatin was sonicated with a Bioruptor Diagenode Sonicator and immunoprecipitated overnight on a wheel at 4°C with 4µg Lamin A/C antibody (anti Lamin A/C (636), from Santa Cruz Biotechnology, sc-7292 x), 5µg histone modification antibodies (anti-H3K4me3, from Active Motif, 39159; anti-H3K27me3, from Abcam, ab6002; anti-H3K9 me3, from Abcam, ab8898) and anti-Suz12 antibodies (Cell Signaling, #3737) at 1:100. The bound complexes were washed with Mixed Micelle buffer (20mM Tris-HCl pH 8.0, 150mM NaCl, 5mM EDTA pH 8.0, 5% Sucrose, 1% Triton X-100, 0.2% SDS), Buffer 500 (50mM Hepes, 0.1% deoxycholic acid, 1% Triton X-100, 500mM NaCl, 1mM EDTA pH 8.0), and LiCl buffer (10mM Tris-HCl, pH 8.0, 0.5% deoxycholic acid, 0.5% NP40, 250mM LiCl, 1mM EDTA pH 8.0). Cross-linking was reversed by incubation at 65°C overnight in elution buffer (100mM $NaHCO_3$, 1%SDS), and DNA was extracted from Dynabead Protein G (Life Technologies) using the standard phenol/chloroform protocol and then resuspended in 20µl of ddH20. qPCR was performed in triplicate using SYBR Select Master Mix for CFX. Ct values were calculated by appropriated software and relative enrichment was calculated as ChIP/input ratio. Primer sequences are given in Supplementary Table 6.

PAT-ChIP. Two 10µm sections of formal-fixed, paraffin-embedded (FFEP) heart tissue were used as starting material. The procedure was adapted from Fanelli et al.[23] as follows. Paraffin was efficiently removed with fresh Histolemon solution (Carlo Erba). Subsequently, Histolemon was replaced with sequential 1ml washes of 100%, 95%, 70%, 50%, 20% ethanol and ddH_2O. For the chromatin extraction step, sections were resuspended in 500 µl of Lysis Buffer (10 mM Tris-HCl pH 7.4, 150 mM NaCl, 3 mM $CaCl_2$, 2 mM $MgCl_2$, 0.5% Tween 20, 10 µg ml^{-1} RNAse A, 0.1 mM PMSF). The lysates were then resuspended in 200 µl of Digestion Buffer. Samples were sonicated four times with a 3-mm microtip probe at 25 W for 30 seconds on ice with a Branson 250. 1 U of

micrococcal nuclease (USB, #70196Y) was added to every 10μg of preheated chromatin (3 minutes at 37°C) and incubated at 37°C for 1 minute. The digestion was stopped by addition of 0.5M EDTA to a final concentration of 12.5 μM. The sections were then resuspended in 200 μl of Extraction Buffer (10 mM Tris-HCl pH7.4, 150 mM NaCl, 3mM $CaCl_2$, 2 mM $MgCl_2$, 0.1% SDS, 0.1 mM PMSF). Samples were resonicated six times with a 3-mm microtip probe at 30 W for 20 seconds on ice. After the centrifugation step the supernatant containing purified chromatin was collected and stored in a clean 1.5 ml Eppendorf tube. The amount of chromatin was checked with Qubit (Thermo Fisher Scientific).

For the immunoprecipitation step, 3μg of ChIP-grade antibody was added to 1μg of tissue chromatin in 500 μl of Incubation Buffer (20mM Tris-HCl pH 7.4, 5mM EDTA, 50mM NaCl, 0.1 mM PMSF) and incubated in rotation (15 rpm) overnight at +4°C. The bound complexes were washed twice with 1 ml of ice-cold Washing Buffer A (20mM Tris-HCl pH 7.4, 5mM EDTA, 50mM NaCl) and 8 ml of the same buffer (containing Triton X-100) were added. After the centrifugation step, the pellet was washed with 10 ml of ice-cold Washing Buffer B (50 mM Tris-HCl pH 7.4, 10mM EDTA, 100mM NaCl) and 10ml of ice-cold Washing Buffer C (50mM Tris-HCl pH 7.4, 10mM EDTA, 150 mM NaCl). The pellet was resuspended in 1 ml of ice-cold Washing Buffer C (without Triton X-100) and transferred to a clean 1.5 ml Eppendorf tube. The bound chromatin was eluted by addition of 300–500 μl 1% SDS in TE. 0.2 M NaCl was added to all samples (input and bound fractions) and samples incubated at 65°C overnight to reverse formaldehyde crosslinks. The next day, 10mM EDTA was added to all samples to a final concentration with 50mM Tris-HCl pH 6.5, 100 μg/ml Proteinase K and incubated at 45°C for 3 hours. The DNA was extracted from protein G-Sepharose (50% slurry, Zymed) with phenol-chloroform. qPCR was performed in triplicate using SYBR Select Master Mix for CFX. Ct values were calculated by appropriated software and relative enrichment was calculated as ChIP/input ratio. Primer sequences are summarized in Supplementary Table 6.

Supplementary References

1. Roncarati, R. *et al.* Doubly heterozygous LMNA and TTN mutations revealed by exome sequencing in a severe form of dilated cardiomyopathy. *Eur J Hum Genet* **21,** 1105–1111 (2013).
2. Sommer, C. A. *et al.* Induced pluripotent stem cell generation using a single lentiviral stem cell cassette. *Stem Cells* **27,** 543–549 (2009).
3. Di Pasquale, E. *et al.* CaMKII inhibition rectifies arrhythmic phenotype in a patient-specific model of catecholaminergic polymorphic ventricular tachycardia. *Cell Death Dis* **4,** e843 (2013).
4. Paulis, M. *et al.* A pre-screening FISH-based method to detect CRISPR/Cas9 off-targets in mouse embryonic stem cells. *Sci Rep* **5,** 12327 (2015).
5. Nakahama, H. & Di Pasquale, E. Generation of Cardiomyocytes from Pluripotent Stem Cells. *Methods Mol Biol* **1353,** 181–190 (2016).
6. Lodola, F. *et al.* Adeno-associated virus-mediated CASQ2 delivery rescues phenotypic alterations in a patient-specific model of recessive catecholaminergic polymorphic ventricular tachycardia. *Cell Death Dis* **7,** e2393 (2016).
7. Weidmann, S. The effect of the cardiac membrane potential on the rapid availability of the sodium-carrying system. *J Physiol* **127,** 213–224 (1955).
8. Miragoli, M., Gaudesius, G. & Rohr, S. Electrotonic modulation of cardiac impulse conduction by myofibroblasts. *Circ Res* **98,** 801–810 (2006).
9. Ma, J. *et al.* High purity human-induced pluripotent stem cell-derived cardiomyocytes: electrophysiological properties of action potentials and ionic currents. *Am J Physiol Hear. Circ Physiol* **301,** H2006-17 (2011).
10. Doss, M. X. *et al.* Maximum diastolic potential of human induced pluripotent stem cell-derived cardiomyocytes depends critically on I(Kr). *PLoS One* **7,** e40288 (2012).

11. Miragoli, M., Salvarani, N. & Rohr, S. Myofibroblasts induce ectopic activity in cardiac tissue. *Circ Res* **101,** 755–758 (2007).

12. Katzung, B. G. Effects of extracellular calcium and sodium on depolarization-induced automaticity in guinea pig papillary muscle. *Circ Res* **37,** 118–127 (1975).

13. Kleber, A. G. & Rudy, Y. Basic mechanisms of cardiac impulse propagation and associated arrhythmias. *Physiol. Rev.* **84,** 431–488 (2004).

14. Satin, J. *et al.* Mechanism of spontaneous excitability in human embryonic stem cell derived cardiomyocytes. *J Physiol* **559,** 479–496 (2004).

15. Bett, G. C. *et al.* Electronic 'expression' of the inward rectifier in cardiocytes derived from human-induced pluripotent stem cells. *Hear. Rhythm* **10,** 1903–1910 (2013).

16. Hoekstra, M., Mummery, C. L., Wilde, A. A., Bezzina, C. R. & Verkerk, A. O. Induced pluripotent stem cell derived cardiomyocytes as models for cardiac arrhythmias. *Front Physiol* **3,** 346 (2012).

17. Zhang, J. *et al.* Functional cardiomyocytes derived from human induced pluripotent stem cells. *Circ Res* **104,** e30-41 (2009).

18. Slaymaker, I. M. *et al.* Rationally engineered Cas9 nucleases with improved specificity. *Science (80-.).* **351,** 84–88 (2016).

19. Ran, F. A. *et al.* Genome engineering using the CRISPR-Cas9 system. *Nat Protoc* **8,** 2281–2308 (2013).

20. Marullo, F. *et al.* Nucleoplasmic Lamin A/C and Polycomb group of proteins: An evolutionarily conserved interplay. *Nucleus* **7,** 103–111 (2016).

21. Whalen, K., Reitzel, A. M. & Hamdoun, A. Actin polymerization controls the activation of multidrug efflux at fertilization by translocation and fine-scale positioning of ABCB1 on microvilli. *Mol Biol Cell* **23,** 3663–3672 (2012).

22. Paulis, M. *et al.* Chromosome transplantation as a novel approach for correcting complex genomic disorders. *Oncotarget* **6,** 35218–35230 (2015).

23. Fanelli, M. *et al.* Pathology tissue-chromatin immunoprecipitation, coupled with high-

throughput sequencing, allows the epigenetic profiling of patient samples. *Proc Natl Acad Sci U S A* **107,** 21535–21540 (2010).
