# Supplementary material for: The K219T-Lamin mutation induces conduction defects through epigenetic inhibition of SCN5A in human cardiac laminopathy
Source: Nat Commun. 2019 May 22;10:2267. doi: 10.1038/s41467-019-09929-w (PMC6531493; doi:10.1038/s41467-019-09929-w)
Supplement: Supplementary file 3 — Reporting Summary [file 41467_2019_9929_MOESM3_ESM.pdf]

nature research

Corresponding author(s): Gianluigi Condorelli and Elisa Di Pasquale

Last updated by author(s): Feb 22, 2019

# Reporting Summary

Nature Research wishes to improve the reproducibility of the work that we publish. This form provides structure for consistency and transparency in reporting. For further information on Nature Research policies, see Authors & Referees and the Editorial Policy Checklist.

## Statistics

For all statistical analyses, confirm that the following items are present in the figure legend, table legend, main text, or Methods section.

| n/a | Confirmed | |
|---|---|---|
| ☐ | ☒ | The exact sample size (*n*) for each experimental group/condition, given as a discrete number and unit of measurement |
| ☐ | ☒ | A statement on whether measurements were taken from distinct samples or whether the same sample was measured repeatedly |
| ☐ | ☒ | The statistical test(s) used AND whether they are one- or two-sided<br>*Only common tests should be described solely by name; describe more complex techniques in the Methods section.* |
| ☒ | ☐ | A description of all covariates tested |
| ☐ | ☒ | A description of any assumptions or corrections, such as tests of normality and adjustment for multiple comparisons |
| ☐ | ☒ | A full description of the statistical parameters including central tendency (e.g. means) or other basic estimates (e.g. regression coefficient) AND variation (e.g. standard deviation) or associated estimates of uncertainty (e.g. confidence intervals) |
| ☐ | ☒ | For null hypothesis testing, the test statistic (e.g. *F*, *t*, *r*) with confidence intervals, effect sizes, degrees of freedom and *P* value noted<br>*Give P values as exact values whenever suitable.* |
| ☒ | ☐ | For Bayesian analysis, information on the choice of priors and Markov chain Monte Carlo settings |
| ☒ | ☐ | For hierarchical and complex designs, identification of the appropriate level for tests and full reporting of outcomes |
| ☒ | ☐ | Estimates of effect sizes (e.g. Cohen's *d*, Pearson's *r*), indicating how they were calculated |

*Our web collection on statistics for biologists contains articles on many of the points above.*

## Software and code

Policy information about availability of computer code

| | |
|---|---|
| Data collection | For flow cytometry, acquisitions were performed on FACS LSRFortessa; FACS Sorting was performed on FACS AriaIII. Images have been acquired using Olympus FV1000 and Leica TCS SP8 STED 3X. For electrophysiology studies, data are collected using pClamp10.6 |
| Data analysis | FACS acquisition were analysed using DIVA Software; for imaging, analyses were performed using ImageJ Fiji and Imaris software (Bitplane). Analysis of electrophysiological recording were perfomed using Clampfit10.6. |

For manuscripts utilizing custom algorithms or software that are central to the research but not yet described in published literature, software must be made available to editors/reviewers. We strongly encourage code deposition in a community repository (e.g. GitHub). See the Nature Research guidelines for submitting code & software for further information.

## Data

Policy information about availability of data

All manuscripts must include a data availability statement. This statement should provide the following information, where applicable:

- Accession codes, unique identifiers, or web links for publicly available datasets
- A list of figures that have associated raw data
- A description of any restrictions on data availability

Data in this study are available from the corresponding authors upon request

# Field-specific reporting

Please select the one below that is the best fit for your research. If you are not sure, read the appropriate sections before making your selection.

☒ Life sciences ☐ Behavioural & social sciences ☐ Ecological, evolutionary & environmental sciences



October 2018

# Life sciences study design

All studies must disclose on these points even when the disclosure is negative.

| | |
|---|---|
| Sample size | For the experiments we employed CMs derived from 2 independent iPSC clones per subject, and data presented as a mean value. Results were considere significant when $p<0.05$ (2-tailed unpaired t-test or two-way Anova) |
| Data exclusions | No data were excluded |
| Replication | Experiments has been performed at least in triplicate and pooled for the analysis unless otherwise stated |
| Randomization | n/a |
| Blinding | 3D-FISH analysis was performed in a blinded fashion. For acquisition of other data, blinding was not performed it was not necessary |

# Reporting for specific materials, systems and methods

We require information from authors about some types of materials, experimental systems and methods used in many studies. Here, indicate whether each material, system or method listed is relevant to your study. If you are not sure if a list item applies to your research, read the appropriate section before selecting a response.

## Materials & experimental systems

| n/a | Involved in the study | |
|---|---|---|
| ☐ | ☒ | Antibodies |
| ☐ | ☒ | Eukaryotic cell lines |
| ☒ | ☐ | Palaeontology |
| ☐ | ☒ | Animals and other organisms |
| ☐ | ☒ | Human research participants |
| ☒ | ☐ | Clinical data |

## Methods

| n/a | Involved in the study | |
|---|---|---|
| ☒ | ☐ | ChIP-seq |
| ☐ | ☒ | Flow cytometry |
| ☒ | ☐ | MRI-based neuroimaging |

## Antibodies

| | |
|---|---|
| Antibodies used | Western Blot: anti-Nav1.5 1:1000 from Cell Signaling, D9J7S; anti-β-actin 1:2000 from Santa Cruz, sc1615.<br>Co-IP: Lamin A/C from Santa Cruz mAb sc-7292-x, anti-Ezh2 from ACTIVE MOTIF mAb catalogue #39875, AC22 clone<br>Immunofluorescence: anti-Nav1.5 channel (anti-rabbit from Alomone, #ASC005; 1:100) and anti-α-sarcomeric actinin (Abcam EA-53 ab9465, 1:100), Alexa Fluor 555-coniugated goat anti-rabbit, 1:500; Alexa Fluor 488-coniugated goat anti-mouse, 1:500, both from Molecular Probes.<br>STED-super resuolution microscopy: anti- Lamin A/C (anti-mouse, from Santa Cruz 7292-X, 1:250) and anti-Suz12 (anti-rabbit from Cell Signaling #3737, 1:250), Abberior STAR-RED and STAR 580-conjugated secondary antibodies.<br>ChIP: anti Lamin A/C (636), from Santa Cruz Biotechnology, sc-7292 x, (anti-H3K4me3, from Active Motif, 39159; anti-H3K27me3, from Abcam, ab6002; anti-H3K9 me3 from Abcam, ab8898, and anti-Suz12 antibodies (Cell Signaling, #3737).<br>3D-FISH: anti Lamin A/C (636), from Santa Cruz Biotechnology, sc-7292 x |
| Validation | All antibodies used in this study were validated by the manufacturer. |

## Eukaryotic cell lines

Policy information about cell lines

| | |
|---|---|
| Cell line source(s) | Cells used in this studies are iPSC lines that we generated from somatic cells of human subjects. We have used two independent lines per subject (2 CNTR and 3 LMNA). |
| Authentication | Lines have been vaidated through extensive characterization: pluripitency, developmental potential, genome stability. |
| Mycoplasma contamination | All generated lines have been tested for micoplasma contamination and resulted negative |
| Commonly misidentified lines<br>(See ICLAC register) | n/a |

## Animals and other organisms

Policy information about studies involving animals; ARRIVE guidelines recommended for reporting animal research

| | |
|---|---|
| Laboratory animals | six-weeks old NOD:SCID mice (Charles River Laboratories) |
| Wild animals | The study did not include wild animals |
| Field-collected samples | The study did not involve samples collected from the field |
| Ethics oversight | The procedure was approved by the Italian Ministry of Health (137/2012-B) |

Note that full information on the approval of the study protocol must also be provided in the manuscript.

## Human research participants

Policy information about studies involving human research participants

| | |
|---|---|
| Population characteristics | Human participant were limited to two gropus, patients carrying the K219T or R190W mutations and controls (without the mutation-healthy). We did not perfomed any clinical study on those. Additional clinical data are in the Supplementary Table 1 |
| Recruitment | Recruitment was carried out by collaborating medical doctors on the basis of the phenotype (LMNA-dependent cardiomyopathy) |
| Ethics oversight | The study was approved by the review boards of three clinical entities: Humanitas Research Hospital (ID:1215), Verona AOUI University Hospital. (ID:765cesc)), and Ospedali Riuniti di Trieste (ID: 74/2015). |

Note that full information on the approval of the study protocol must also be provided in the manuscript.

## Flow Cytometry

### Plots

Confirm that:

☒ The axis labels state the marker and fluorochrome used (e.g. CD4-FITC).

☒ The axis scales are clearly visible. Include numbers along axes only for bottom left plot of group (a 'group' is an analysis of identical markers).

☒ All plots are contour plots with outliers or pseudocolor plots.

☒ A numerical value for number of cells or percentage (with statistics) is provided.

### Methodology

| | |
|---|---|
| Sample preparation | CMs were enzymatically dissociated into single cells, fixed in 1% paraformaldehyde and permeabilized; staining and detection were then performed using mouse monoclonal α-sarcomeric actinin antibody (1:400 from Abcam, Cambridge, UK) and goat anti-mouseAlexa-647-conjugated antibody (1:500 from Molecular Probes, Thermo Scientific), respectively.<br>Dead cells were quantified and excluded from the analysis using the LIVE/DEAD fixable aqua stain kit (Molecular Probes, Thermo Fisher Scientific) |
| Instrument | FACS LSRFortessa flow cytometer (BD Bioscience, San Jose, CA, USA)<br>FACS Aria III for cell sorting |
| Software | DIVA software (BD Pharmingen, San Diego, CA, USA) |
| Cell population abundance | Described in the manuscript - Supplementary Fig. 4 |
| Gating strategy | Provided in the Supplementary Figure 4 and in the Supplementary Fig 15 |

☒ Tick this box to confirm that a figure exemplifying the gating strategy is provided in the Supplementary Information.
