# Supplementary material for: The K219T-Lamin mutation induces conduction defects through epigenetic inhibition of SCN5A in human cardiac laminopathy
Source: Nat Commun. 2019 May 22;10:2267. doi: 10.1038/s41467-019-09929-w (PMC6531493; doi:10.1038/s41467-019-09929-w)
Supplement: Supplementary file 4 — Description of Additional Supplementary Files [file 41467_2019_9929_MOESM4_ESM.pdf]

Description of Additional Supplementary Files

File Name: Supplementary Movie 1
Description: Contracting CMs differentiated from K219T-iPSCs

File Name: Supplementary Movie 2
Description: Contracting CMs differentiated from CNTR-iPSCs
